# Combating Fuel Biocontamination: Tailored Antimicrobial Peptides and an Innovative Delivery Strategy

**DOI:** 10.1021/acsabm.5c00474

**Published:** 2025-05-22

**Authors:** Swagata Das, Uttam Pal, Tanusri Saha-Dasgupta, Susanna Leong

**Affiliations:** † Food, Chemical and Biotechnology Cluster, 372463 Singapore Institute of Technology, Singapore 138683, Singapore; ‡ Technical Research Centre, S. N. Bose National Centre for Basic Sciences, Kolkata 700106, India; § S. N. Bose National Centre for Basic Sciences, Kolkata 700106, India

**Keywords:** fuel biocontamination, microbial clusters, antimicrobial peptides, peptide delivery, broad-spectrum antimicrobials

## Abstract

Microbial invasion and subsequent fuel biocontamination have long posed significant challenges, leading to a significant infrastructural damage. The lack of systematic data on the correlation between environmental parameters and microbial growth has hampered the development of targeted solutions to date. To address this challenge, this study reports a targeted strategy to inactivate and control the proliferation of commonly identified fuel-contaminating microbial clusters through the development of synthetic peptides that can be delivered directly to fuel samples. From a library of short peptides which was designed based on the indolicidin template peptides, three unique sequences were found to have good broad-spectrum activity toward a range of microbes such as *Bacillus*, *Sphingomonas*, and *Hormoconis*, with P17, showing the highest killing potential. The structural analyses of the peptides based on circular dichroism spectroscopy revealed the helical propensity of the peptides in SDS micelles and a random flexible structure in solution. The peptides showed stability under biological conditions and minimal cytotoxicity against mammalian cells. This study presents an innovative method to effectively address fuel biocontamination using short peptides coupled with a potentially scalable protocol to administer the peptides to fuel samples.

## Graphical abstract

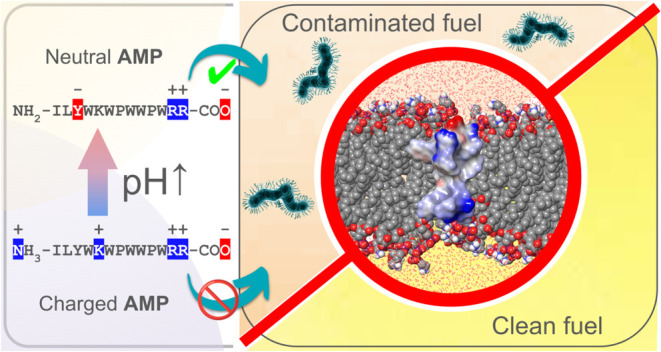

## Introduction

Biodegradation of fuel remains a significant challenge in many industrial sectors. The absence of systematic and comprehensive documentation of fuel-degrading microbes has resulted in generalized attempts to address this problem, often with limited success. Microbial contamination of fuel was first documented by Miyoshi in 1895,^1,2^ where bacteria caused accelerated corrosion and increased sulfur content in aircraft fuel storage systems.^3,4^ By the 1930s, 11 microorganisms had been identified as capable of utilizing hydrocarbons as a sole carbon source, suggesting the proliferation of bacteria and fungi in fuel and the deposition of their biomass in fuel tanks as a key driver for microbial contamination.^5^ The US Air Force (USAF) observed microbial contamination in aviation gasoline and kerosene as early as 1950s, where sludge deposition was found in the water-bottoms of underground fuel storage tanks which was characterized by the presence of viable bacteria and their metabolic byproducts. Reports in the later years indicated that aviation fuel and diesel are more prone to microbial attack compared to gasoline due to the inherent lower water holding capacity of gasoline.^4,6–11^ The water layer at the bottom of the fuel tanks/reservoirs serves as a habitat for microbes, with the primary sources of microbial entry being vents, leaks, water seepage, transfer piping, cross contamination, and leakage of underground tanks as well as deposition of water through condensation. Seawater seepage introduces organic and inorganic nutrients along with a heavy load of microbial inoculum that can easily adapt to the natural microenvironment.^12^ According to storage specifications, the allowable water and sediment content in fuels is limited to 0.5% by volume, meaning a 10,000 m^3^ fuel tank can hold up to 2 m^3^ of water, which is a significant quantity for fostering microbial growth.^13–15^

The most common method of controlling microbial contamination is the addition of chemical fuel biocides such as Kathon FP1.5 and Biobor JF which can inhibit microbial growth.^16^ However, chemical biocides have several drawbacks such as higher corrosion risks, health and environmental concerns, reduced fuel quality or stability, and potential microbial resistance. Biocide partitioning is also a major challenge in nonpolar fuel environments. Confronted by these challenges, other mitigation strategies are needed to effectively combat fuel contamination.

This study explores the use of antimicrobial peptides (AMPs) to tackle fuel-contaminating microbes. AMPs are short chains of amino acids with specific hydrophobicity and charge capable of targeting single or multiple microbes such as bacteria and fungi.^17–19^ Although they are known as robust antimicrobial agents, the use of AMPs in tackling fuel contamination is limited to date. In this first of its kind study, several short peptides with novel sequences were designed to target microorganisms that are prevalent in fuel systems. Several of these designed peptides were found to inhibit biofilm-forming bacteria such as *Pseudomonas, Sphingomonas*, and *Roseomonas*. Besides demonstrating minimal hemolytic activity, the peptides are resistant to protease degradation and stable in the presence of high salt concentrations, making them suitable candidates to be explored further to address fuel contamination. A method to readily administer the peptides in fuel systems was also established, which suggests scalability of this proposed control strategy.

### Chemicals

All of the chemicals used are of research grade purchased from Sigma or Merck, if not mentioned otherwise. The microbial culture media were purchased from BioBasic and/or Himedia. The peptides used in the study were synthesized by GL Biochem (Shanghai) Ltd. using the standard F-moc solid-phase synthesis method. Initial peptide stock solutions (2 mg/mL) were prepared with an acetonitrile: water (1:4 v/v) mixture. Required concentrations of the peptide solutions were prepared in phosphate buffer from the stock, each was centrifuged, and the supernatant was filtered using a 0.22 μm filter before being used in the studies reported. For minimal inhibitory concentration (MIC) assays, the peptide was diluted to 30 to 60 μM from a peptide stock concentration of 500 μM. The microbial strains were procured from ATCC. Sheep red blood cells (RBCs) which were used for the hemolysis assay were procured from Thermo Fischer Scientific (cat no. SR0051B).

### Microbial Clusters

The microbial clusters selected for use in this study (Table 1) were based on their prevalence in contributing to fuel contamination as reported in the literature.^7,11,20–28^ The list of microbial clusters chosen included all of the abundant bacteria phyla, viz. Firmicutes, Actinobacteria, and Proteobacteria comprising both the Gram-positive and Gram-negative bacteria as well as the most abundant fungal phylum prevalent in fuel biocontamination, i.e., Ascomycota.

**Table 1:** List of the Microbial Clusters Used in This Study

microbial clusters	phylum		ATCC no.
bacteria			
Gram-positive	firmicutes	*Macrococcus caseolyticus* (MC)	ATCC 13548
		*Bacillus licheniformis* (BL)	ATCC 14580
	actinobacteria	*Dermacoccus nishinomiyaensis* (DN)	ATCC 29093
		*Kocuria rosea* (KR)	ATCC 186
		*Micrococcus yunnanensis* (MY)	ATCC 12698
Gram-negative	proteobacteria	*Roseomonas mucosa* (RM)	ATCC BAA-692
		*Sphingomonas paucimobilis* (SP)	
		*Pseudomonas aureginosa* (PA)	ATCC 39327
fungi			
	ascomycota	*Hormoconis resinae* (HR)	ATCC 20267

## Methods

### Peptide Design

Template-assisted peptide design strategy was adopted to develop novel peptide sequences for this study. Indolicidins, which are a broad-spectrum peptide targeting several microbes, were chosen as the template to design novel sequences of short peptides. Two peptide design strategies were employed; (A) Systematic single-residue modifications were made based on the hydrophobicity index, where the substituted amino acids were chosen based on their higher hydrophobicity index according to a descending order of hydrophobicity, i.e., W, F, Y, I, L, V, P, A, M, C, H, T, S, N, Q, D, E, K, R, G^29–34^ and (B) Replacement of the Pro-residue preceding the “PWWP” signature motif in indolicidins with other hydrophobic amino acids. The latter approach was inspired by findings where Ala-substitution of Pro-residues in CP10A, an indolicidin derivative, had significantly improved MIC values against Gram-positive bacteria.^35^ In this study, the Pro-residue at the third position was substituted with amino acids having a higher hydrophobicity index than proline with the aim to achieve peptides with higher hydrophobicity and hence antimicrobial activity potential. In both the strategies, the replacement of one amino acid with another retains the peptides’ chemical properties and keeps its overall charge (+3) consistent.

The purity of the peptides was ascertained by high-performance liquid chromatography (HPLC) using Polaris 5 C18-A 25 mm × 4.6 mm reverse phase Zorbax columns from Agilent Technologies. The buffer system contained 0.1% (v/v) TFA in 100% water and 0.1% (v/v) TFA in 100% acetonitrile. Each peptide sample contained 300 μM peptides dissolved in an acetonitrile–water (1:4) mixture. A constant gradient of the two solvent systems was used to generate the chromatogram for 30 min after sample injection.

### Antimicrobial Assays

#### Antibacterial Assay

All of the bacterial species were grown overnight at 37 °C in LB broth. 10^7^ CFU/mL of cells were resuspended in fresh culture and grown until the mid log phase.^36,37^ The bacterial cells were adjusted to a concentration of 5 × 10^5^ CFU/mL to quantify the MIC. Equal volumes of the respective bacterial cultures were incubated with the serially diluted peptides in 96-well microtiter plate and incubated overnight at 37 °C in ambient air. Microorganisms without peptides were treated as the positive control, while uninoculated broth was used as the negative control. The MIC was taken as the lowest peptide concentration at which observable bacteria growth was inhibited, as measured by a microplate reader (Biotek Synergy H1 microplate reader). Experiments were performed in triplicates, and the mean MIC values were determined. A similar study was performed to understand the effect of peptide purity, i.e., ranging from 70 to 95%, on the peptide MIC.

#### Antifungal Assay

The same experimental procedures that were conducted for the antibacterial assay were used to determine the MIC of the peptides against the fungal strain, *H. resinae*.

#### Hemolysis Assay

The RBCs were centrifuged at 1000g for 15 min at 4 °C to remove the buffy coat and washed 3 times with PBS. 100 μL of 4% (v/v) RBC was suspended in PBS and added into sterilized 96-well plates. 100 μL of peptide solution was added to the suspension of RBCs by serial dilution starting from 200 μM to 3.175 μM and incubated overnight at 37 °C. Sample absorbance at 545 nm was measured to determine hemoglobin release after centrifuging the plate at 600 g for 10 min. The percentage of cell lysis was calculated using the following formula (eq 1).^32,36^

1 $$\%\;\mathrm{lysis}=\frac{{\mathrm{OD}}_{545}\;\left(\mathrm{test}\right)-{\mathrm{OD}}_{545}\;\left(\mathrm{blank}\right)}{{\mathrm{OD}}_{545}\;\left(\mathrm{total}\;\mathrm{lysis}\right)-{\mathrm{OD}}_{545}\;\left(\mathrm{blank}\right)}\times 100$$

### Peptide Stability Studies

The stability of the peptides against one Gram-positive (*B. licheniformis*) and Gram-negative (*S. paucimobilis*) bacteria was assessed by determining their MIC in chymotrypsin (protease), serum, and salt (NaCl). The experiments were adopted from Selvarajan et.al.^37^ The fold change in MIC with respect to the control was calculated and determined for each peptide against the microbial clusters.

#### Susceptibility of Peptides to Protease Degradation and Serum

To determine the susceptibility of the engineered peptides, which are abundant in Trp, to protease degradation, chymotrypsin, which cleaves Tyr, Phe, and Trp at physiological pH was chosen as the model protease. The protocol described by Wang et.al. (2014) was used with slight modifications.^32^ Peptides were incubated with chymotrypsin at a molar ratio of 40:1 (peptide: protease) in 10 mM PBS buffer (pH7.4) at 37 °C, and samples were analyzed after 2 h. The enzyme reaction was terminated by incubating the samples at 80 °C for 10 min. The incubated peptide mixtures were then serially diluted in 96-well plates to determine the MIC values of the microbial strains.

The stability of the peptides in the presence of the serum was tested using a 10% fetal bovine serum (heat inactivated). MIC was determined for all of the peptides against the microbial clusters incubated with the serum at 37 °C.

#### Salt Sensitivity Assay

The effect of salt (150 mM NaCl) on the MIC of the peptides was studied after overnight incubation at 37 °C. We also studied the effects of 600 mM NaCl (*in vitro*) and natural seawater (NSW) on the MICs of the engineered peptides.

### Assessing the Hydrocarbon Uptake Capacity of Selected Microbial Clusters and the Effectiveness of Peptides on the Spiked Fuel Samples

To study the partitioning of microbes in fuel tanks, filter-sterilized fuel was mixed with BH medium in a 1:1 (v/v) ratio. The inoculum was added to the aqueous phase at the bottom to simulate growth in the aqueous layer of fuel tanks and incubated aerobically at 30 °C for 30 days. Visible growth was assessed, and aliquots from both the aqueous and fuel phases were plated on nutrient agar plates to evaluate microbial growth. Peptides were initially added to the aqueous layer of microbially spiked fuel samples for screening the effectiveness in the fuel environment. The study was further extended over a longer duration, and incubated under aerobic conditions at 30 °C for 30 days. Microbial growth was monitored at regular intervals by plating aliquots from the aqueous layer on the nutrient agar plates. The negative control comprised fuel which had no inoculum, while the positive control comprised inoculum without AMPs.

### Circular Dichroism (CD) Studies of the Peptides

Far-UV CD spectra of each peptide candidate were measured using a J-1500 spectrophotometer (Jasco) equipped with a Peltier type temperature control system. All of the spectra were measured at 20 °C over the range of 160–260 nm (3 scans, 1 nm bandwidth, 20 nm/min scan speed, 2 s response time) using a 0.02 cm path length cell. For each sample, three scans were averaged, and the buffer background was subtracted. All samples were prepared by diluting a stock peptide solution (1 mg/mL) to a final peptide concentration of 0.125 mg/mL, followed by the addition of SDS (0.016–16 mM, i.e., below and above cmc; cmc ≈ 4 mM) for peptide solutions comprising SDS.^38^ Additionally, SDS solutions were prepared to a concentration of 200 mM to study their effect on the structural transitions of the peptides. SDS measurements below and above cmc enable investigating the effects of SDS acting both as (a) a denaturation agent (below cmc) and (b) a simple membrane model (above cmc).^38^ Structural changes of the peptides in bicarbonate buffer (0.1 M) used for peptide delivery studies were also studied in solutions prepared in a 1:1 (v/v).

### Molecular Dynamics (MD) Simulation Studies to Determine Mode of Action of the Peptides

MD simulations were carried out using Desmond^39^ as implemented in Schrodinger Maestro (Academic Release 2020-3). The peptide molecules were placed in the center of a cubic periodic boundary box with an edge length of 5 nm so that the periodic images are at least 2 nm apart. Force field specific atom types and partial charges were generated for the peptides. Interatomic interactions were defined by the OPLS (optimized potentials for liquid simulations) force field^40,41^ following a previously published protocol.^42,43^ Two different solvent systems were used, i.e., preoptimized SPC (simple point-charge) water environment and octanol (a common fuel component) to mimic the experimental conditions. Charged systems (at neutral pH) were neutralized with counterions. The temperature of the system was slowly increased from 0 to 300 K through a series of short (∼10 ps) simulations using the Nosé-Hoover chain thermostat method keeping the volume and number of atoms constant (NVT ensemble). Finally, 10 ns of simulation was run, keeping the pressure constant at 1 bar using the Martyna–Tobias–Klein barostat algorithm (NPT ensemble). The system state evolves over time using the reference system propagator algorithm (RESPA).^44^ Long-range Coulombic interaction was treated with the particle mesh Ewald (PME) method. For short-range Coulombic interactions, the cutoff radius was 9 Å. Coordinates of the system were saved at 10 ps interval. Secondary structural properties were assigned by DSSP,^45^ and solvation energies were obtained from the simulation trajectories using Schrodinger Maestro (Academic Release 2020-4).

### Isoelectric Point Determination, Partition Coefficient Calculation and *In Vitro* Peptide Delivery Administration

#### Isoelectric point and partition coefficient determination

Partition coefficients and isoelectric points of the peptides were computed based on their chemical structure using Chemaxon Marvin (https://www.chemaxon.com). When a molecule is ionizable at a given pH, it forms a hydrophilic anion or cation and does not dissolve in organic solvents. The standard partition coefficient of ionized and un-ionized species calculated from the molecular structure is based largely on the atomic log P increments given in an earlier study.^46^ The extent of ionization at a given pH is obtained from the predicted p*K*_a_ of the molecule.

#### Establishment of an *In Vitro* Peptide Delivery Method

P17 was selected as the model peptide for studies to establish a viable delivery method. The peptide was solubilized in a bicarbonate buffer (pH 10) and administered to the top layer of the spiked fuel samples. The control setup was administered with the peptide dissolved in PBS buffer (pH 7). The peptide concentration was aligned with its MIC value against a microbial cluster. Two representative Gram-positive strains (*B. licheniformis* and *M. caseolyticus*) and Gram-negative strains (*P. aeruginosa* and *S. paucimobilis*) were used for this study. The negative control did not comprise any inoculum or peptide, while the positive control comprised the inoculum but no peptides. Details of the experimental setup are listed in Table 2.

**Table 2:** Experimental Setup Used in the Peptide Delivery Studies Involving Bicarbonate Buffer^a^

S/N	sample composition		peptide solubilization buffer	location of peptide delivery
1 (a)	BH + JF + BL inoculum (10^5^ CFU/mL)	control (+)		
1 (b)	BH + JF + MC inoculum (10^5^ CFU/mL)			
1 (c)	BH + JF + PA inoculum (10^5^ CFU/mL)			
1 (d)	BH + JF + SP inoculum (10^5^ CFU/mL)			
2	BH + JF	control (−)		
3 (a)	BH + JF + BL inoculum (10^5^ CFU/mL)	+P17	bicarbonate buffer system (pH 10)	fuel layer
3 (b)	BH + JF + MC inoculum (10^5^ CFU/mL)			
3 (c)	BH + JF + PA inoculum (10^5^ CFU/mL)			
3 (d)	BH + JF + SP inoculum (10^5^ CFU/mL)			
4 (a)	BH + JF + BL inoculum (10^5^ CFU/mL)	+P17	PBS buffer system (pH 7.4)	fuel layer
4 (b)	BH + JF + MC inoculum (10^5^ CFU/mL)			
4 (c)	BH + JF + PA inoculum (10^5^ CFU/mL)			
4 (d)	BH + JF + SP inoculum (10^5^ CFU/mL)			

a BH, Bushnell Hass medium; JF, Jet fuel; BL, *Bacillus licheniformis*; MC, *Macrococcus caseolyticus*; PA, *Pseudomonas aeruginosa*; SP, *Sphingomonas paucimobilis*.

## Results and Discussion

### Design and Antimicrobial Studies of Novel Short Peptides for Fuel Decontamination

This study aims to design robust broad-spectrum peptides that are capable of targeting fuel-contaminating microbial clusters with minimal cytotoxic effects. The peptides were designed based on the template of a naturally occurring broad-spectrum AMP, indolicidins. Indolicidins are 13 amino acid long cationic peptides with abundant Trp (39%) and Pro (23%) residues.^35^ The signature motif, “PWWP,” of indolicidins enables flexibility in solution to adapt up to 8 stereoisomeric forms, thus rendering its broad-spectrum activity.^33^ The peptide design framework was focused on retaining the parent peptide’s overall native charge while one amino acid residue was being substituted at a time. Thirteen novel peptide sequences were generated using this method, and an additional 5 peptide analogs were generated by substituting the Pro-residue ahead of the “PWWP” motif with other hydrophobic amino acids, i.e., Trp, Phe, Tyr, Ile, and Leu (Table 3). The purity of the peptides was ascertained through mass spectrometry and HPLC (Figures S1–S4).

**Table 3:** Library of Peptides Designed Based on the Indolicidin Template

(A) substituting 1 amino acid at a time, with an amino acid of similar chemical property													
indolicidin (template)/ P1	Ile	Leu	Pro	Trp	Lys	Trp	Pro	Trp	Trp	Pro	Trp	Arg	Arg
P2	Ile	Leu	Pro	Trp	Lys	Trp	Pro	Trp	Trp	Pro	Trp	Arg	***Lys***
P3	Ile	Leu	Pro	Trp	Lys	Trp	Pro	Trp	Trp	Pro	Trp	***Lys***	Arg
P4	Ile	Leu	Pro	Trp	Lys	Trp	Pro	Trp	Trp	Pro	***Phe***	Arg	Arg
P5	Ile	Leu	Pro	Trp	Lys	Trp	Pro	Trp	Trp	***Val***	Trp	Arg	Arg
P6	Ile	Leu	Pro	Trp	Lys	Trp	Pro	Trp	***Phe***	Pro	Trp	Arg	Arg
P7	Ile	Leu	Pro	Trp	Lys	Trp	Pro	***Phe***	Trp	Pro	Trp	Arg	Arg
P8	Ile	Leu	Pro	Trp	Lys	Trp	***Val***	Trp	Trp	Pro	Trp	Arg	Arg
P9	Ile	Leu	Pro	Trp	Lys	***Phe***	Pro	Trp	Trp	Pro	Trp	Arg	Arg
P10	Ile	Leu	Pro	Trp	***Arg***	Trp	Pro	Trp	Trp	Pro	Trp	Arg	Arg
P11	Ile	Leu	Pro	***Phe***	Lys	Trp	Pro	Trp	Trp	Pro	Trp	Arg	Arg
P12	Ile	Leu	***Val***	Trp	Lys	Trp	Pro	Trp	Trp	Pro	Trp	Arg	Arg
P13	Ile	***Ile***	Pro	Trp	Lys	Trp	Pro	Trp	Trp	Pro	Trp	Arg	Arg
P14	***Tyr***	Leu	Pro	Trp	Lys	Trp	Pro	Trp	Trp	Pro	Trp	Arg	Arg

(B) replacing the pro-residue in the third position with amino acids of higher hydrophobicity than Pro													
indolicidin (template)	Ile	Leu	Pro	Trp	Lys	Trp	Pro	Trp	Trp	Pro	Trp	Arg	Arg
P15	Ile	Leu	***Trp***	Trp	Lys	Trp	Pro	Trp	Trp	Pro	Trp	Arg	Arg
P16	Ile	Leu	***Phe***	Trp	Lys	Trp	Pro	Trp	Trp	Pro	Trp	Arg	Arg
P17	Ile	Leu	***Tyr***	Trp	Lys	Trp	Pro	Trp	Trp	Pro	Trp	Arg	Arg
P18	Ile	Leu	***Ile***	Trp	Lys	Trp	Pro	Trp	Trp	Pro	Trp	Arg	Arg
P19	Ile	Leu	***Leu***	Trp	Lys	Trp	Pro	Trp	Trp	Pro	Trp	Arg	Arg

These engineered peptides were tested for their antimicrobial efficacy against selected microbial clusters, which are commonly reported to be present in contaminated fuel systems. Initial screening of the peptides’ ability to inhibit microbial growth was based on the disc diffusion assay (data not shown), followed by the determination of MIC values. Most peptide analogs showed higher antimicrobial efficacy against Gram-positive bacteria compared to Gram-negative bacteria and fungi (Table S1), which might be due to the varying structural complexities of the microbial membranes.^19,47,48^ Among all of the engineered peptide, P17 showed the highest broad-spectrum activity, demonstrating effectiveness against Gram-positive bacteria (*Bacillus licheniformis*, *Macrococcus caseolyticus*, *Dermacoccus nishinomiyaensis*, *Kocuria rosea*, *Micrococcus yunnanensis*), Gram-negative bacteria (*Pseudomonas aeruginosa*, *Sphingomonas paucimobilis*, and *Roseomonas mucosa*), and fungi (*Hormoconis resinae*). Other peptides with a reasonable broad-spectrum profile include P3 and P11 (Table 4). We also extended our findings to additional fungal clusters namely, *Aspergillus niger*, *Candida albicans*, *Alternaria alternata*, *Aureobasidium pullulans*, and *Penicillium oxalicum* to test efficacy of the peptides against these. The peptides showed considerable efficacy against fungal clusters, but not as compared to their efficacy against bacterial clusters (data not shown). Moreover, P3 and P11 showed better results against the fungal proliferations of *Aspergillus niger* and *Penicillium oxalicum*. P17 could restrict the growth of *A. alternata*, whereas the growth of *Candida* could only be restricted by the template peptide, P1. The effect of peptide purity on efficacy against Gram-positive bacteria, *B. licheniformis* and Gram-negative bacteria, *S. paucimobilis* was also studied, where lower peptide purity generally compromised the efficacy of the peptides except for P17, which retained its efficacy at lower purity ranges (data not shown).

**Table 4:** Minimum Inhibitory Concentration (MIC), Cytotoxicity, and Hydrophobicity of the Engineered Peptides^a^

	MIC (μM)										
	bacteria								fungi		
	Gram-negative			Gram-positive							
	proteobacteria			actino-bacteria			firmicutes		ascomycota		
peptides	PA	RM	SP	MY	DN	KR	MC	BL	HR	HL_50_ (μM)	*R*_t_ (min)
P1	–	–	0.9	0.9	0.9	1.9	0.9	0.9	–	≥200	13.067
P3	–	1.9	1.9	0.9	0.9	–	0.9	1.9	–	≥200	12.731
P11	1.9	–	1.9	0.9	0.9	–	0.9	1.9	–	≥200	12.875
P17	3.8	–	0.9	0.9	0.9	1.9	1.9	0.9	3.8	≥200	12.625

a HL_50_ (μM), conc. to reach 50% cell lysis (sheep blood RBCs); *R*_t_, retention time of peptide on HPLC column as a function of peptide hydrophobicity. “–” depicts no detectable activity of the peptides against the respective microbes.

Recognizing that contaminated fuel systems harbor diverse microbial communities, we tested the efficacy of the peptides on lab-grown *in vitro* microbial cocultures comprising both bacterial and mycelial cells (see Figure S5 and Tables S2 and S3).

### Cytotoxicity and Stability Studies of the Engineered Peptides

The cytotoxicity of the engineered peptides against mammalian red blood cells was studied. All AMPs showed negligible cytotoxicity against mammalian RBCs up to a peptide concentration of 200 μM, where minimal (≤4%) lysis was observed (Table 4). P17, the most broad-spectrum peptide from the library, which contained 5 aromatic amino acids, i.e., 4 Trp residues and 1 Tyr residue, along with Pro-residues, also showed minimal cytotoxicity. These results corroborate with the high antimicrobial potency of dCATH from ducks which showed no hemolytic activity attributable to the abundance of Trp residues.^49^ Tyr is reported to play an important role in anchoring the peptides to the microbial membranes and ensuring structural stability by stabilizing hydrogen bonds and aromatic interactions, thereby maximizing the antimicrobial effectiveness.^50–54^ Pro-residues, on the other hand, play a very crucial role in native indolicidins^33,34,55^ as well as in other AMPs like Bac7 1-23 ^56^ rendering extensive antimicrobial property. The retention of the signature indolicidin motif, “PWWP,” and the presence of Tyr along with the presence of Trp residues are, therefore, hypothesized to contribute to the high efficacy observed in P17, as the PWWP domain is highly associated with DNA binding in nuclear proteins such as hepatoma-derived growth factors (HDGF) and DNA methyltransferases.^57,58^ Although the exact role of this motif in indolicidins is yet to be clearly established, “PWWP” is believed to contribute to DNA-binding and form a helical turn structure, responsible for extensive antimicrobial property.^59^

The functional efficacy of AMPs is often impaired by susceptibility to physiological degradations. Major factors that contribute to these degradations include proteolytic enzymes, salts, and serum.^60^ For fuel contamination, the presence of salt due to seawater contamination is a common challenge. In this study, the stability of the engineered peptides was evaluated by testing their antimicrobial efficacy against a representative Gram-positive (*B. subtilis*) and Gram-negative (*S. paucimobilis*) strain in the presence of (a) protease, i.e., chymotrypsin, that specifically cleaves at Trp, Tyr, and Phe which are abundant in these peptides, (b) salt, and (c) fetal bovine serum (FBS). Upon incubating the peptide with chymotrypsin for 2 h, the engineered peptides showed no increase in the native MIC value, which indicates their proteolytic stability (Figure S6a,b). The engineered peptides also retained their antimicrobial activity in the presence of 150 mM NaCl, 600 mM NaCl, and natural seawater, as well as the serum where only a slight increase in MIC values were observed against Gram-negative bacteria (although still well within the acceptable range of ≤4 μM).

### Efficacy Studies of the Engineered Peptides in Fuel Environments

After establishing the hydrocarbon utilizing property of the microorganisms (see Figure S7), the efficacy of the engineered peptides in simulated fuel storage environments (1:1 v/v aqueous phase:fuel phase) spiked with selected bacterial clusters was studied. An initial screening of the all of the active peptides (Table S4) were performed to understand the efficacy. The best peptides (Table 4) were selected based on the activity in fuel and were further tested for efficacy in fuel for a 30-day duration. The engineered peptides were administered at their respective MICs to the aqueous layer of simulated fuel samples, and samples from both the aqueous and solvent (fuel) layers were removed at intervals of 7, 14, 21, and 30 days and plated on LBA plates to assess cell viability (Table S5). The engineered peptides inhibited the growth of most bacterial clusters present in the spiked fuel samples except for *P. aeruginosa*, which exhibited a resurgence after 21 days. This resurgence is hypothesized to be due to dormant cells escaping the peptides, a common survival strategy in *Pseudomonas* associated with biofilm formation.^61^ P11 and P17 were particularly effective in suppressing *P. aeruginosa* growth for nearly 20 days. *S. paucimobilis*, another biofilm-forming Gram-negative bacterium, was inhibited by the peptides over 30 days. *H. resinae*, a fungal species, was effectively suppressed for 15 days, after which slow growth resumed in the aqueous phase (data not shown). This pattern is consistent with reports that microbial proliferation in contaminated fuel is often contained within the water layer and interface of the aqueous-solvent layer, with microbes drawing nutrients from the fuel phase.^1,62^ Overall, the administered peptides demonstrated good efficacy in the samples in simulated fuel environments.

### Structural Studies of the Engineered Peptide(s) Using Circular Dichroism Spectroscopy and Molecular Dynamics Simulations to Determine the Possible Mechanism of Action

Indolicidins are known to achieve their antimicrobial activity through different mechanisms including membrane disruption and pore formation.^34,35,59,63^ Membrane pore formation by small helical peptides is one of the most common strategies to exert antimicrobial activities in most AMPs.^19,52^ The model engineered peptide, P17, in this study shows broad-spectrum activity against different microbial clusters, but the exact mechanism of action remains unexamined. The shift in the structural motifs of short AMPs when interacting with the microbial membrane and other cellular components is attributed to the intricate changes and rearrangements in the secondary structure elements, often coined as the “structure–activity relationship (SAR).” Short cationic peptides tend to assume a loosely structured random coil conformation in solution and adopt structured conformations on binding microbial membranes.^48^ The electrostatic attraction between the positively charged AMPs and the negatively charged bacterial membranes results in the insertion of the hydrophobic ends of the peptide into the lipid bilayer leading to membrane disorganization by inducing toroidal pore (i.e., wormhole), barrel-stave, or the carpet model phenomenon, and eventually, cell death.^64,65^

The CD spectra of the engineered peptides in PBS buffer showed random disordered structures, with a single dominant negative peak between 202 and 208 nm. Other than the random structures, some rather unique combinations of extended helical structures like π-helices, 3_10_ helices, and helix turns may also have contributed to the structural composition of these short AMPs. Native indolicidin also showed a negative peak around 205 nm, which is usually designated to mostly unordered structures (Figure 1a,b). Other than the classical helical motifs, polyproline II (PPII)-like helices^35,57^ are very common in small peptides compared to bigger globular proteins, playing a crucial role in the interaction with biological macromolecules. Natively disordered proteins or short peptides have a very high abundance of PPII motifs, characterized as the negative peak around 205 nm in the CD spectrum, which was observed in the engineered peptides. Other observable extended helical motifs such as 3_10_ helices, which are generally identified by a negative bulge at 208 nm and negligible to no negative bulge at 222–225 nm, were also observed in the engineered peptides. P17 has a much broader negative trough, i.e., 203–208 nm, which might be due to the presence of extended disordered conformations as well as the presence of bulky aromatic residues (Trp and Tyr) and their interaction with the solvent.

**Figure 1:**
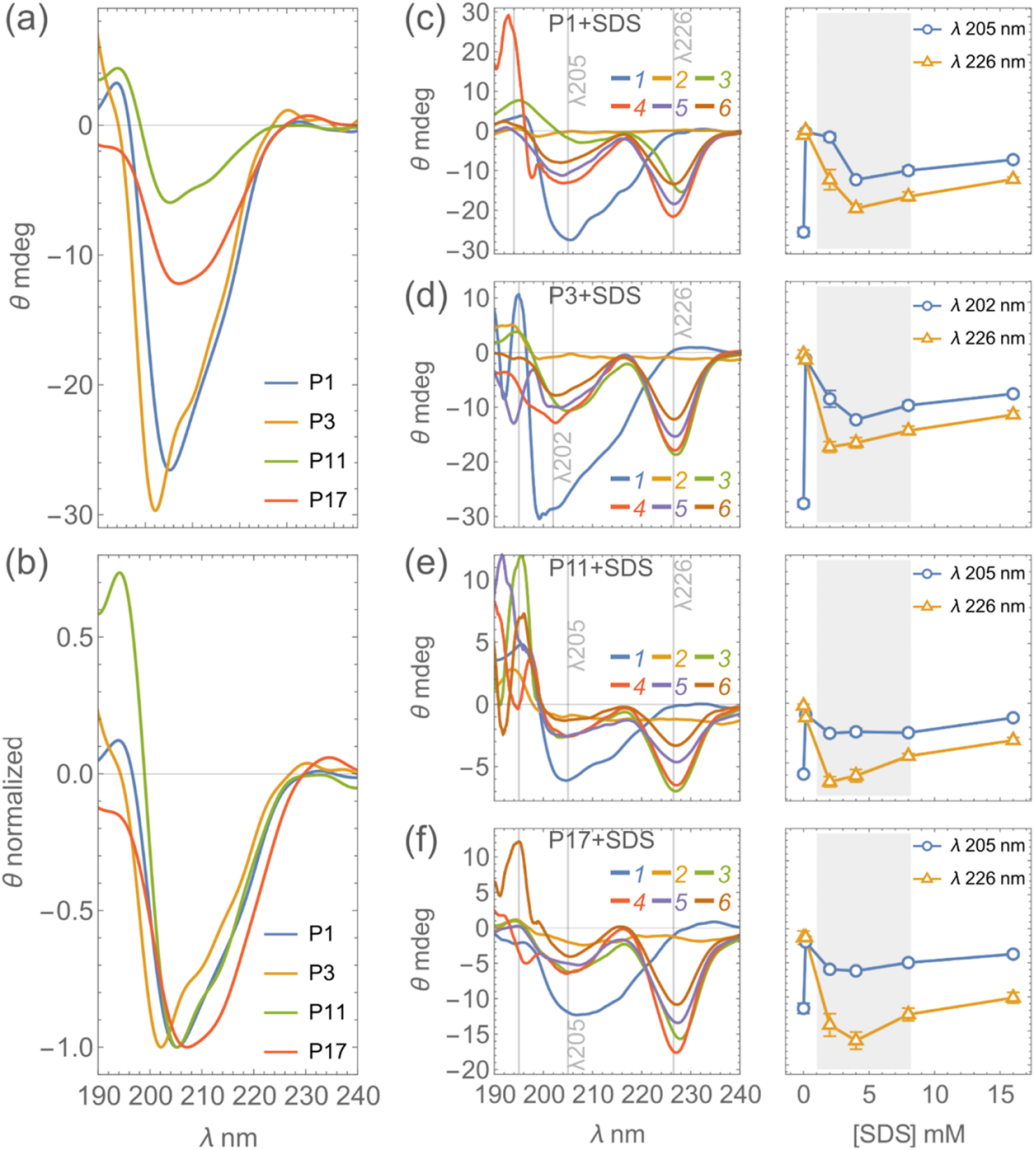
CD spectra of the engineered peptides. (a) CD spectra of P1 (blue), P3 (orange), P11 (green), and P17 (red) in PBS buffer. (b) Normalized CD spectra of the engineered peptides at their respective negative peak minimum. All of the peptides, except P17 registered a negative trough around 201–205 nm. P17 had a broad negative bulge between 203 and 207 nm. (c–f) CD spectra of the engineered peptides in SDS micelles for P1 (c), P3 (d), P11 (e), and P17 (f), respectively. The spectra were recorded in increasing concentrations of SDS ranging from 0.16 to 16 mM (1, blue, native; 2, orange, 0.16 mM SDS; 3, green, 2 mM SDS; 4, red, 4 mM SDS; 5, purple, 8 mM SDS; and 6, brown, 16 mM SDS). Significant spectral transitions were observed in the presence of SDS micelles. Along with the α-helical conformational peaks (∼205 nm), tryptophan signature peaks also overlapped at around 226 nm wavelength. The two signature peak intensities are plotted as a function of SDS concentration on the right panel. Lower concentrations of SDS acted as the denaturant, disrupting the structure. Structural features are most prominent in the intermediated to micellar concentrations of SDS (highlighted in gray) which mimicked membrane models, thus stabilizing the secondary structure.

Significant structural transitions were observed in the presence of the SDS micelles. The critical micellar concentration of SDS varies with several factors, including the temperature and the dissolved ions of the buffer. The CD studies were performed with an increasing SDS concentration ranging from 0.16 to 16 mM. At SDS concentrations of less than 2 mM where SDS is a potential denaturant,^66^ a decrease in peptide helicity due to denaturation of the native peptide was generally observed ((Figure 1c–f, orange line in the spectra denoting 0.16 mM SDS) and Table 5). Stable compact helical structures were observed with an increasing concentration of SDS (Figure 1c–f) aligning with the structural stability in membrane mimicking conditions. The increase in the helical content (to up to almost 58.9% from native 27% in case of P17) indicates that the peptides underwent significant structural rearrangement in the presence of SDS micelles. The presence of the negative trough around 225–230 nm is hypothesized to be due to the mixed contribution from the multiple Trp residues, extended helices (3_10_ helices), and the α helical motifs. The 230 nm Trp band provides indications about the local environment as well as the secondary structure of the peptides/proteins.^67^ When the peptides were incubated in higher concentrations of SDS, i.e., up to 200 mM, increased structural randomness was observed, indicating that the critical micellar concentration plays a significant role in compacting the peptides’ structures, mimicking the impact of the microbial membranes in compacting the peptide structures prior to exerting the function. All of the structural contents in the study were measured using the web-based tool BeStSel (http://bestsel.elte.hu/index.php).

**Table 5:** Helicity Content of the Engineered Peptides in SDS Micelles as Determined from Circular Dichroism (CD) Spectroscopy Studies

	helical content (%) of the peptides			
peptide/buffer composition	P1	P3	P11	P17
PBS buffer	45.7	38.4	14.3	27.1
SDS 0.16 mM	0	0	0	22.5
SDS 2 mM	57.4	55.4	23.5	58.9
SDS 4 mM	63.5	38.1	20.2	43.4
SDS 8 mM	50.1	34.4	26.4	52.2
SDS 16 mM	54	34.6	4.1	37.8

To augment our understanding of structural transitions in the CD spectroscopy results, MD simulations was conducted to understand the secondary structures of P17, which was chosen based on its broad-spectrum efficacy in water and octanol (fuel mimic) environments (Figure S8). The mixed helical fingerprints as observed from the CD spectra can be correlated with the MD simulation findings, showing that the peptide’s secondary structure includes helix turns, α-helices, and 3_10_-helices (Figure S8a). In water, it adopts a loosely helical structure with wider helix turns at the N-terminal and narrower α- and 3_10_ - helices at the C-terminal (Figure S8a–c), whereas in octanol, the peptide’s helicity increases, with dominant α- and 3_10_-helices (Figure S8b). The peptide assumes a globular form in water (Figure S8d) and a rod-like helical conformation in octanol (Figure S8d), resulting in a larger radius of gyration in octanol (Figure S8e). The higher radius of gyration renders a more compact structure, resulting in better function, and can be correlated to the effective control of the fuel-contaminating microbial clusters. MD simulation studies at varying pH shows that at higher pH, the peptide predominantly exhibits narrow 3_10_-helices (Figure S9a), similar to its structure in octanol (Figure S9b). At neutral pH, the peptide carries a net charge of +3, but this becomes zero at its isoelectric point (pI) due to deprotonation. Despite this, the peptide retains surface point charges that influence its structure (Figure S9c). Interestingly, at this neutral charge state, the peptide showed similar structural features in both water and octanol, as reflected in a comparable radius of gyration in both environments (Figure S9d–f).

To assess the membrane disruption potential of peptides, MD simulations were conducted by embedding P17 in a model phospholipid bilayer resembling a microbial membranes. The calculated solvation free energies in the membrane (Table S6) were significantly lower than in water or octanol, indicating a thermodynamically favorable transfer of the peptide from both aqueous and fuel environments to the microbial membrane. This result suggests that peptides in these environments can inhibit microbial growth. Additionally, the peptide is likely to remain in the membrane rather than enter the intracellular fluid, aligning with the mechanism of native indolicidins, which inactivate microbes by disrupting their membranes rather than targeting intracellular components.^55^

To understand P17’s potential for membrane disruption, its structural disposition in the membrane environment was analyzed via MD simulations. P17 predominantly exhibits helix turns and 3_10_ - helices, with the C-terminal favoring a narrow 3_10_ helix conformation (Figure 2a). The peptide length is sufficient to span the membrane width (Figure 2b), and its radius of gyration indicates elongation in the membrane compared to that in octanol or water (Figure 2c). The peptide retains partial charges at both terminal regions (Figure 2d,e), suggesting a mechanism of membrane disruption. The interaction of these charges with the zwitterionic phospholipid head groups (e.g., phosphatidylcholine) can induce toroidal deformation of the membrane, leading to thinning and potential water channel formation, causing membrane leakage (Figure 2e). A close investigation of the lipid conformations surrounding the peptide in the membrane, as obtained from MD simulations, gave a better understanding of the probable membrane disruption mechanism (Figure 3). A cross section of the membrane with embedded peptide, P17, is shown in Figure 3a along with the water distribution on either side of the membrane. As the two terminals of the peptide are charged, they attract water to form funnel-shaped indentations on both sides of the membrane (Figure 3b). Study of the phospholipid head groups also reveals bending (Figure 3c) leading to potential toroidal deformation as postulated in Figure 3c. Mapping of water over the MD trajectory further reveals the thinning of the membrane around the peptide insertion site and formation of a potential water channel (Figure 3d). It is also interesting to note that in the potential energy surface map, one side of the peptide appears to be nonpolar (Figure 3d, last pose), which may allow lateral association of multiple peptides in the fluid membrane leading to the formation of a larger pore complex. All of these findings corroborate the robust efficacies of the identified peptide analogs, using P17 as a model.

**Figure 2:**
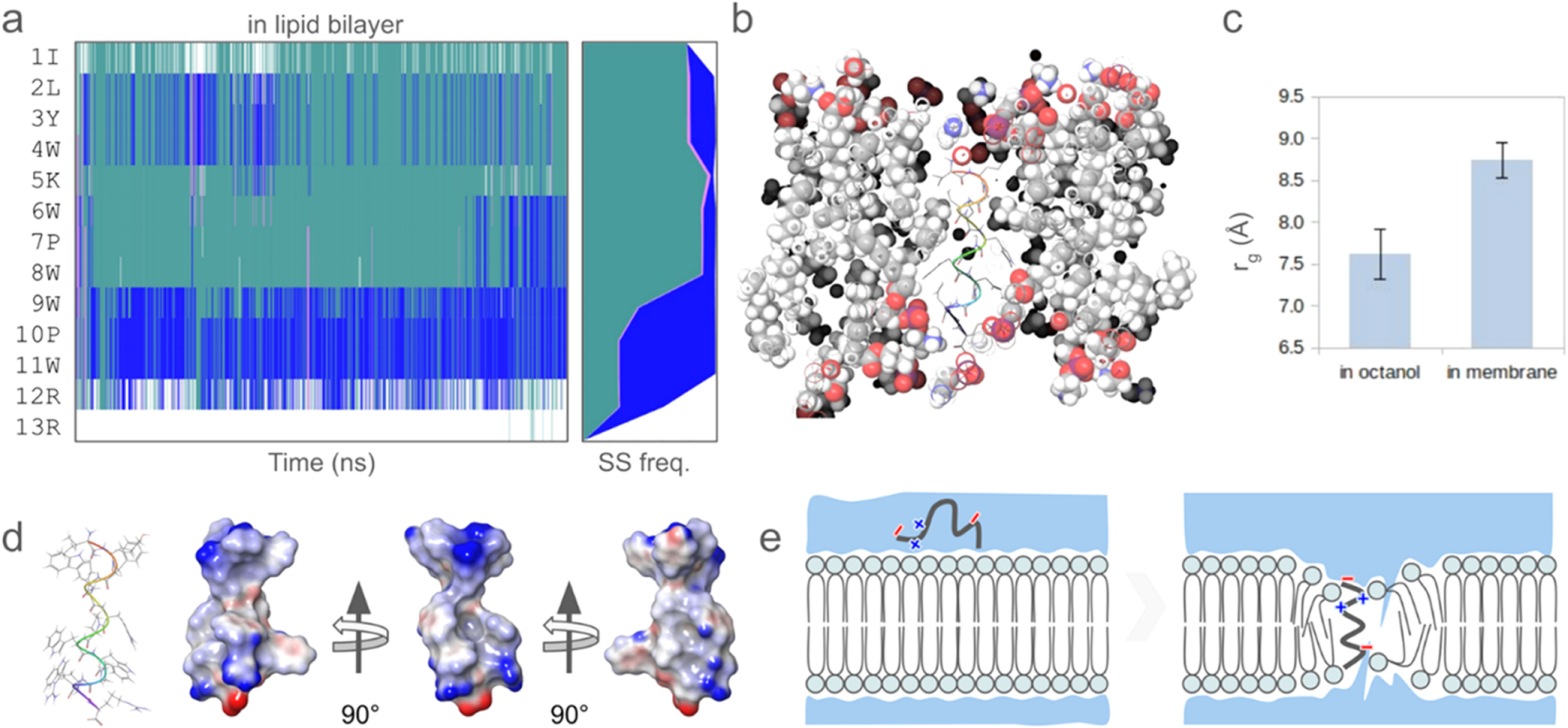
Structure and localization of P17 in lipid bilayer. (a) Residue-wise secondary structural propensities of P17 over 100 ns in phospholipid bilayer. Helix turn (turquoise), and 3_10_ helix (blue) conformations were mainly observed. (b) Cross-section view of the lipid bilayer (space filling with standard color) showing transmembrane helical conformation of P17. (c) Significantly higher radius of gyration of P17 indicates elongated structure. (d) Electrostatic potential map on the surface of P17 showing distribution of partial positive (blue) and negative (red) charges. (e) Schematic representation of membrane disruption by P17. Strong electrostatic interaction between charged amino acid residues with zwitterionic head groups of membrane phospholipids can potentially lead to toroidal deformation of membrane and pore formation.

**Figure 3:**
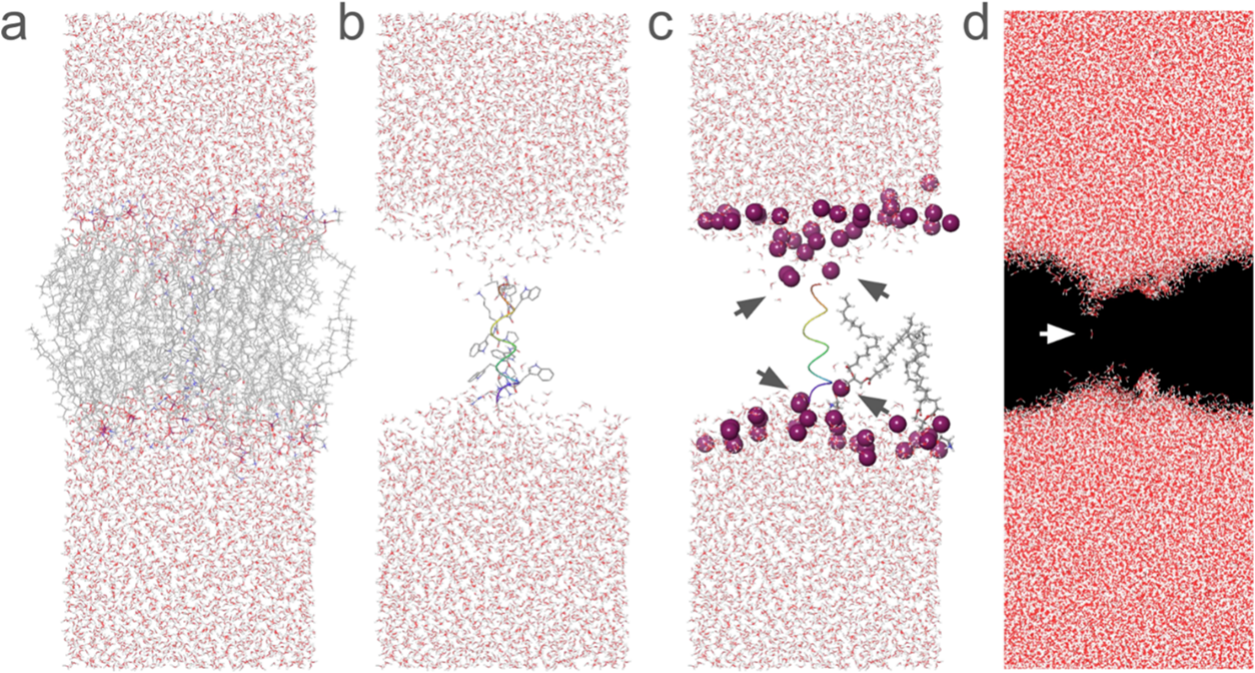
Disruption of lipid bilayer by P17. (a) Snapshot of the molecular dynamics simulation showing the lipid bilayer separating water into two domains. (b) Same frame with the membrane bilayer hidden to give a clear view of P17 in the membrane. Elongated helical structure of P17 spans the thickness of the bilayer and the two terminals of P17 are exposed to the water. (c) Same frame with the phosphorus atoms (purple spheres) of the membrane phospholipids shown. The arrowheads point toward inward bending of polar head groups where the peptide is inserted. Tails of a phospholipid molecule near the peptide and one at a distance are shown highlighting the toroidal deformation in the membrane. (d) Superposition of 100 frames from MD simulation of the same cross section showing only the water distribution. Arrowhead points toward potential water channel formation.

### Partition Coefficient of the Peptides and Development of a Delivery Mechanism of the Peptides to Fuel Samples

Studies were next conducted to evaluate the mechanism of delivery of the engineered peptides into the fuel layer to facilitate the ease of delivery.

#### Thermodynamics of Peptide Delivery from Fuel to an Aqueous Phase

Partition coefficient (log *D*) of the peptides is defined as the ratio of the peptide concentration in a polar (water) and nonpolar (octanol) solvent mixture at equilibrium. It was determined from the chemical composition of the peptide using a machine-learning (ML) model as implemented in ChemAxon (https://www.chemaxon.com).

log *D* is defined as follows:

$$\log D=\log c_{o}/c_{w}=\log c_{o}-\log c_{w}$$

where *c*_o_ is the peptide concentration in octanol and *c*_w_ is the peptide concentration in water.

From the establishment of the peptide’s structural propensities via CD and MD simulations, the thermodynamics of its solvation in polar and nonpolar environments was investigated, as it governs the peptide’s transfer rate between phases (oil to water). Initial log D analysis revealed high water solubility at pH 7.0 but low solubility in octanol, which challenges peptide applications. MD simulations of P17 equilibrated in water and octanol using the OPLS force field was conducted.^40,68,69^ The intermolecular interaction energies of the peptide derived under this force field are listed in Table S7. Energies were derived for both the charged and the neutral configurations of P17 (Figure S9c). There are mostly two types of nonbonded interactions, i.e., Coulomb and van der Waals (vdW). Coulombic and vdW interactions dominate in the polar and nonpolar environments, respectively. The charged peptide with +3 net charge shows highly favorable interactions with water which is reflected in the highly negative solvation free energy (Δ*G*_sol_). However, the solvation free energy of P17 in octanol was found to be positive, indicating unfavorable solvation (Table S7). The peptide with a net +3 charge can remain in octanol only in the presence of counterions (Table S7), which again is an unrealistic situation.

The ionization of peptides and their log D values are pH dependent, whereas when the pH increases, peptides deprotonate and become neutral at pH 10 to 12. From neutral pH (7.4) to pH 9, peptides show greater affinity for water (Table S8), but above pH 9, they show a greater affinity for nonpolar media. Increasing the pH of a peptide solution to around 10 significantly drives peptide partition into the fuel, which can be achieved by solubilizing the peptide in an alkaline buffer. To enhance peptide delivery to fuel, the use of alkaline media was proposed based on pH-dependent partition coefficient calculations. As pH approaches the peptide’s pI, the peptide becomes charge neutral. MD simulations of the neutral P17 peptide in water and octanol (using the OPLS force field) revealed thermodynamically favorable interactions with both solvents (Table S7). The solvation free energies suggest a slight increase in peptide solubility in water and a significant increase in the nonpolar phase. These findings were further validated by conducting *in vitro* delivery of the peptides under an alkaline environment.

#### *In Vitro* Peptide Delivery

To investigate the viability of an alkaline peptide delivery environment, the bicarbonate buffer system (pH 10) was chosen as the peptide solubilization buffer. The structural integrity and antimicrobial efficacy of P17 were retained in the bicarbonate buffer system as determined by CD spectroscopy and MIC determination (data not shown). The peptide in the bicarbonate buffer was administered to the top of the solvent layer of a microbe-spiked fuel sample and monitored for growth over 30 days. The aqueous layer was observed using the naked eye for turbidity at 7-day intervals. In positive controls (with no peptides added), turbidity of the aqueous phase increased over time, with CFU counts reaching approximately as high as 10^9^ CFU/mL by day 7 or 8. Table 6 and Figure 4 show the turbidity and microbial growth in the different samples. All of the studied strains were inhibited up to 30 days in the fuel samples except for *P. aeruginosa*, where it could only be controlled up to 15 days.

**Figure 4:**
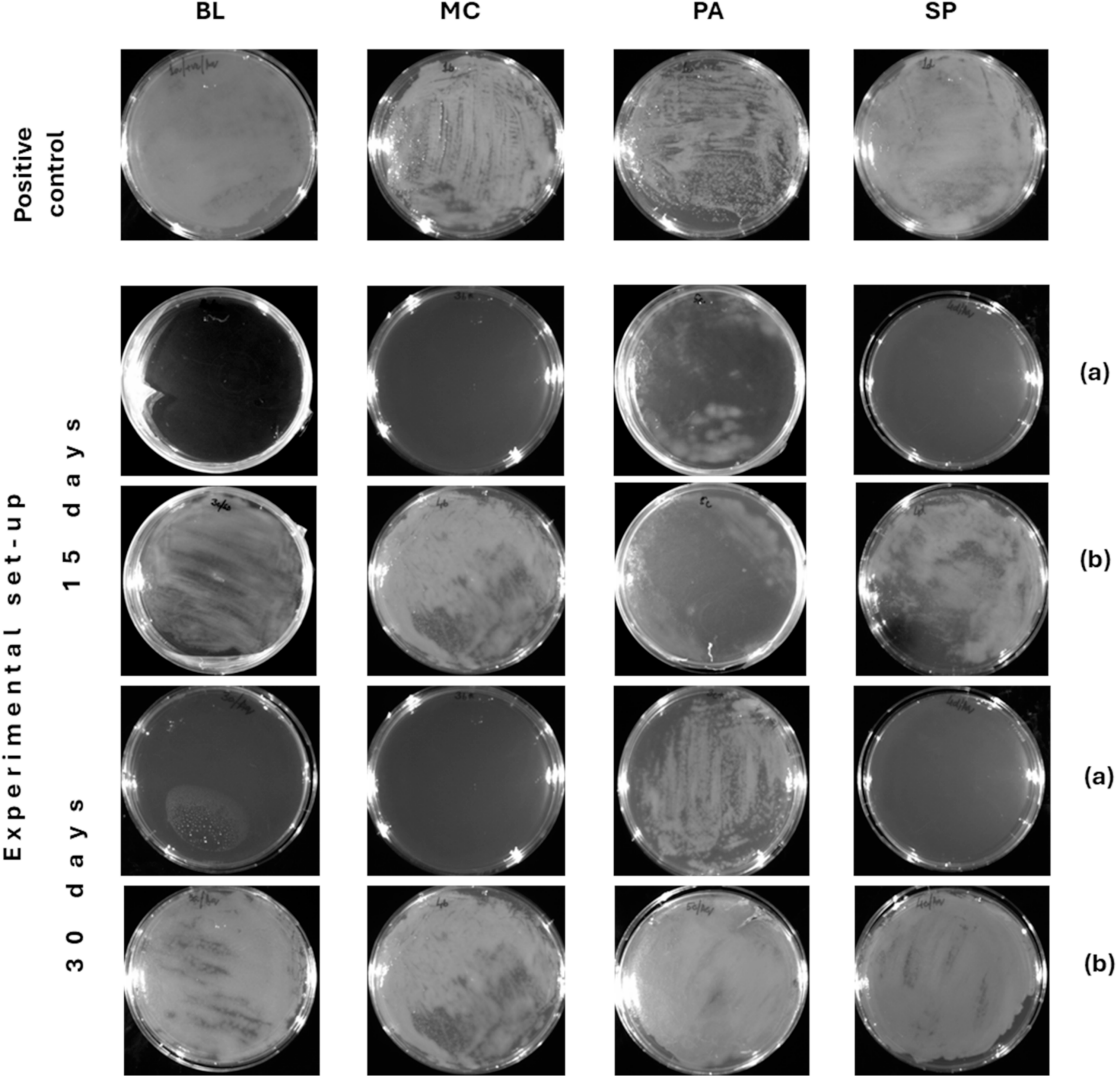
P17 solubilized in the bicarbonate buffer and delivered to the fuel samples. (a) Growth inhibition of *B. licheniformis* (BL), *M. caseolyticus* (MC), *P. aeruginosa* (PA), and *S. paucimobilis* (SP) was observed when P17 in bicarbonate buffer was delivered to the top layer of the microbe-spiked fuel sample. P17 in bicarbonate buffer was able to restrict the growth of all of the bacterial clusters until 30 days, except PA which showed growth after 15 days. (b) P17 administered in PBS buffer, when administered to the top layer of the spiked fuel was unable to inhibit the growth of the microbes, as shown. The positive control was spiked with bacterial inoculum but did not contain any peptides.

**Table 6:** Peptide Inhibition Pattern of the Bacterial Clusters in Bicarbonate and PBS Buffer Systems^a^

					bacterial cluster inhibition	
					no. of incubation days	
S/N	details of the experimental setup		peptide solubilization buffer	location of peptide delivery	15 days	30 days
1 (a)	BH + JF + (BL)	control (+)			+	+
1 (b)	BH + JF + (MC)				+	+
1 (c)	BH + JF + (PA)				+	+
1 (d)	BH + JF + (SP)				+	+
2	BH + JF	control (−)			–	–
3 (a)	BH + JF + (BL)	+P17	bicarbonate buffer system (pH 10)	top layer (in fuel)	–	–
3(b)	BH + JF + (PA)				–	–
3 (c)	BH + JF + (PA)				–	+
3 (d)	BH + JF + (PA)				–	–
4 (a)	BH + JF + (BL)	+P17	PBS buffer system (pH 7.4)	top layer (in fuel)	+	+
4 (b)	BH + JF + (MC)				+	+
4 (c)	BH + JF + (PA)				+	+
4 (d)	BH + JF + (SP)				+	+

a BH, Bushnell Hass medium; JF, Jet fuel; BL, *Bacillus licheniformis*; MC, *Macrococcus caseolyticus*; PA, *Pseudomonas aeruginosa*; SP, *Sphingomonas paucimobilis*. “+” indicates appearance of microbial growth.

When the peptide was solubilized in PBS, inhibition of microbial growth was not achieved, as documented by confluent microbial growth in the aqueous phase. The results of this study point to the viability of using an alkaline solubilization buffer such as the bicarbonate buffer to deliver P17 effectively in fuel systems to kill microbes which typically congregate in the aqueous phase.

## Conclusions

The indolicidin-inspired engineered short peptides developed in this study exhibit significant potential for mitigating microbial contamination in fuel systems. These AMPs are characterized by their high stability and negligible cytotoxicity, making them suitable to counter fuel biocontamination, as well as for other applications. The MICs of the indolicidin-derived peptide analogs are comparable to those of the parent peptide, with promising antimicrobial activity against multiple bacterial strains such as *Macrococcus*, *Dermacoccus*, *Kocuria*, *Roseomonas*, *Sphingomonas, Bacillus*, and *Pseudomonas* as well as the fungal strain, *Hormoconis*. Stability studies show that the engineered peptides consistently suppressed most of the bacterial clusters over a 30-day period, except *Pseudomonas*. These engineered peptides displayed a promising MIC range (≤4 μM) and maintained functional stability for extended durations, with P11 and P17 showing potential antibiofilm properties. *In vitro* and *in silico* structural studies revealed that the peptides underwent significant structural rearrangement to adopt a stable helical conformation during the antimicrobial activity, contrasting with their random coil structure observed in the buffer. An innovative but simple peptide delivery mechanism was developed to demonstrate the ease by which these peptides could be introduced to fuel systems, which paves the way for an alternative solution to chemical-based biocides, which does not induce corrosion or pose any known safety and environmental risks.

## Supplementary Material



## Notes

Additional hydrocarbon utilization methods; effects of the engineered peptides on the spiked fuel samples and screening the best performing peptides based on an in-housing scoring matrix; thermodynamics calculations of P17 in membrane, water, and octanol; and the secondary structural propensities of P17 and charge neutral P17 in water and octanol environment. (PDF)
