# Supplementary material for: Combating Fuel Biocontamination: Tailored Antimicrobial Peptides and an Innovative Delivery Strategy
Source: ACS Appl Bio Mater. 2025 May 22;8(9):7672–83. doi: 10.1021/acsabm.5c00474 (PMC12442081; doi:10.1021/acsabm.5c00474)
Supplement: Supplementary file 1 [file mt5c00474_si_001.pdf]

Supporting Information

Combating Fuel Biocontamination: Tailored Antimicrobial Peptides and an Innovative Delivery Strategy

Swagata Das[1]*, Uttam Pal[2], Tanusri Saha-Dasgupta[3] and Susanna S. J. Leong[1]

[1]Food, Chemical and Biotechnology Cluster, Singapore Institute of Technology, Singapore138683, Singapore; [2]Technical Research Centre, S. N. Bose National Centre for Basic Sciences, Kolkata 700106, India, [3]S. N. Bose National Centre for Basic Sciences, Kolkata 700106, India

Corresponding author: Swagata Das. Email. swagata.das@singaporetech.edu.sg

HPLC and MS data of the engineered peptides.

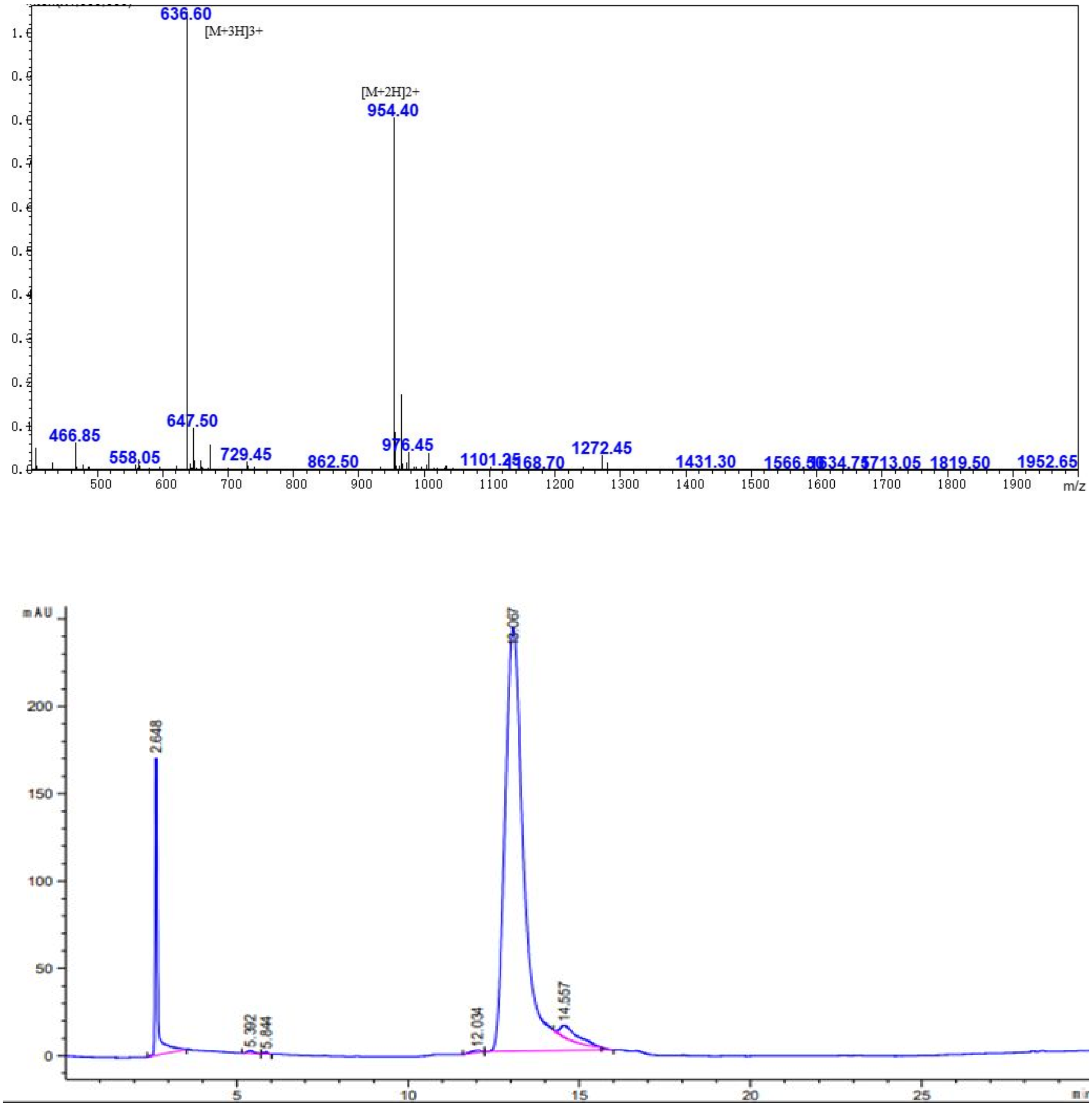

Figure S1. MS spectra (upper panel) and the RP-HPLC chromatogram (lower panel) of purified P1.

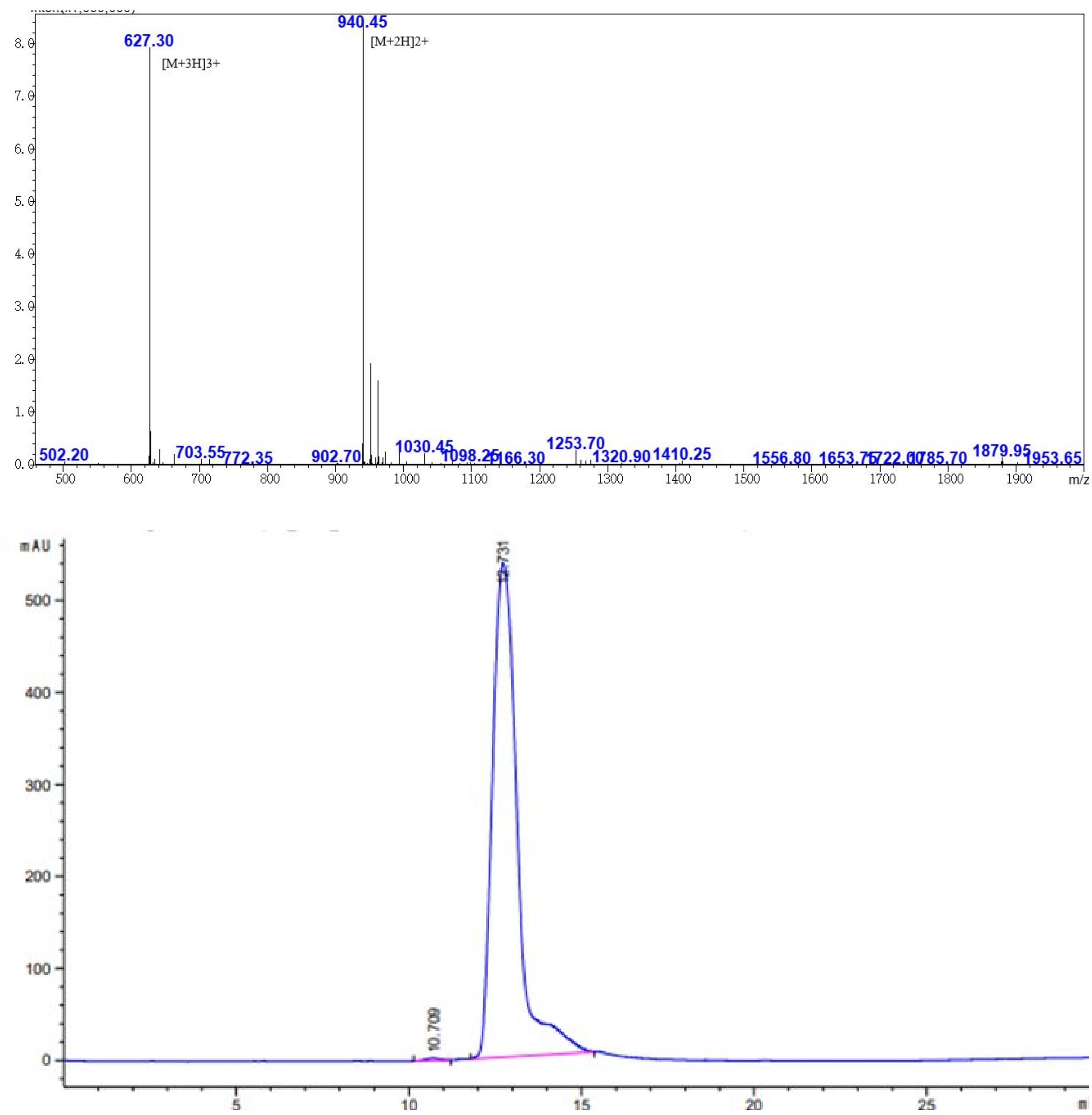

Figure S2. MS spectra (upper panel) and the RP-HPLC chromatogram (lower panel) of purified P3.

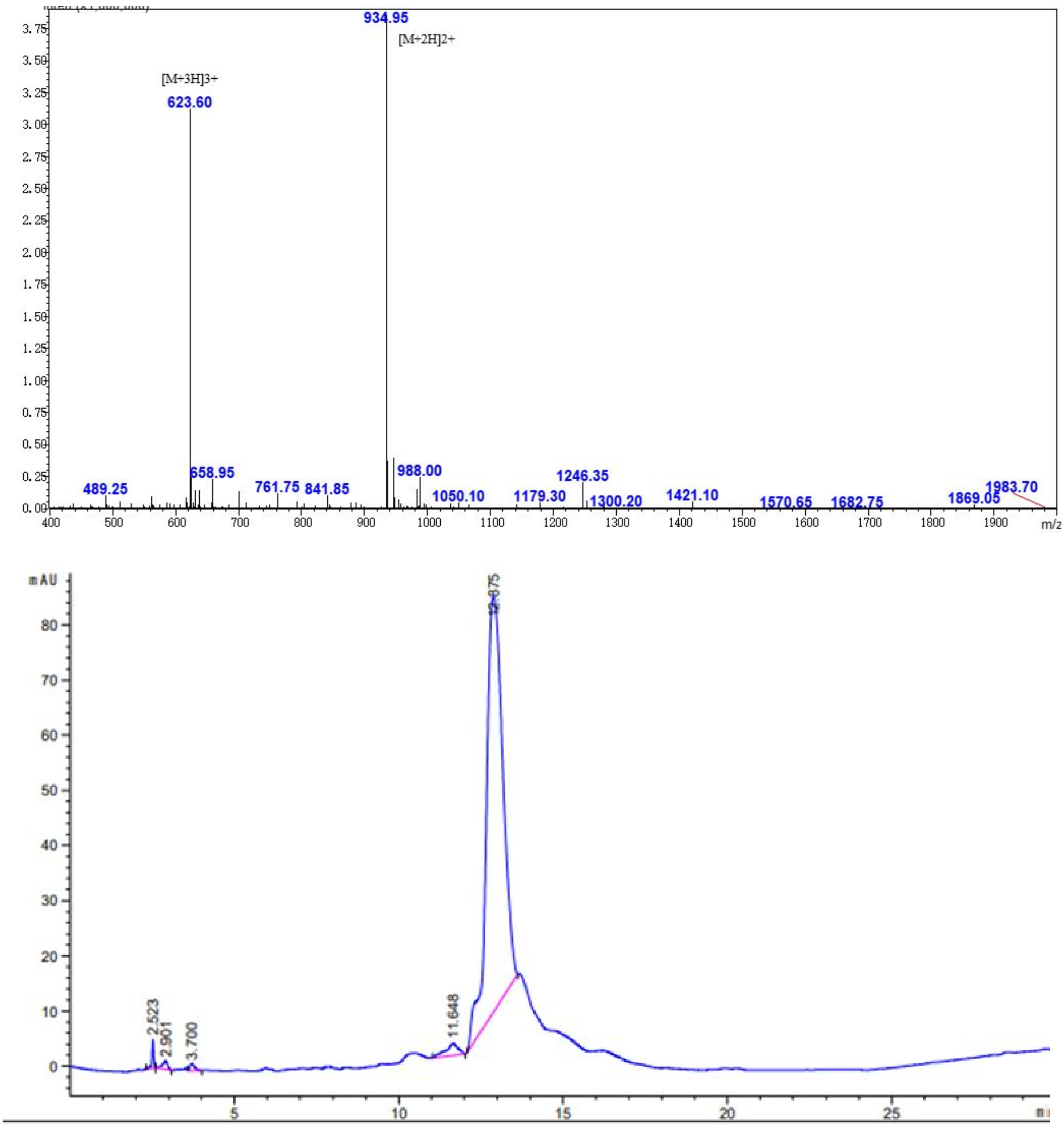

Figure S3. MS spectra (upper panel) and the RP-HPLC chromatogram (lower panel) of purified P11.

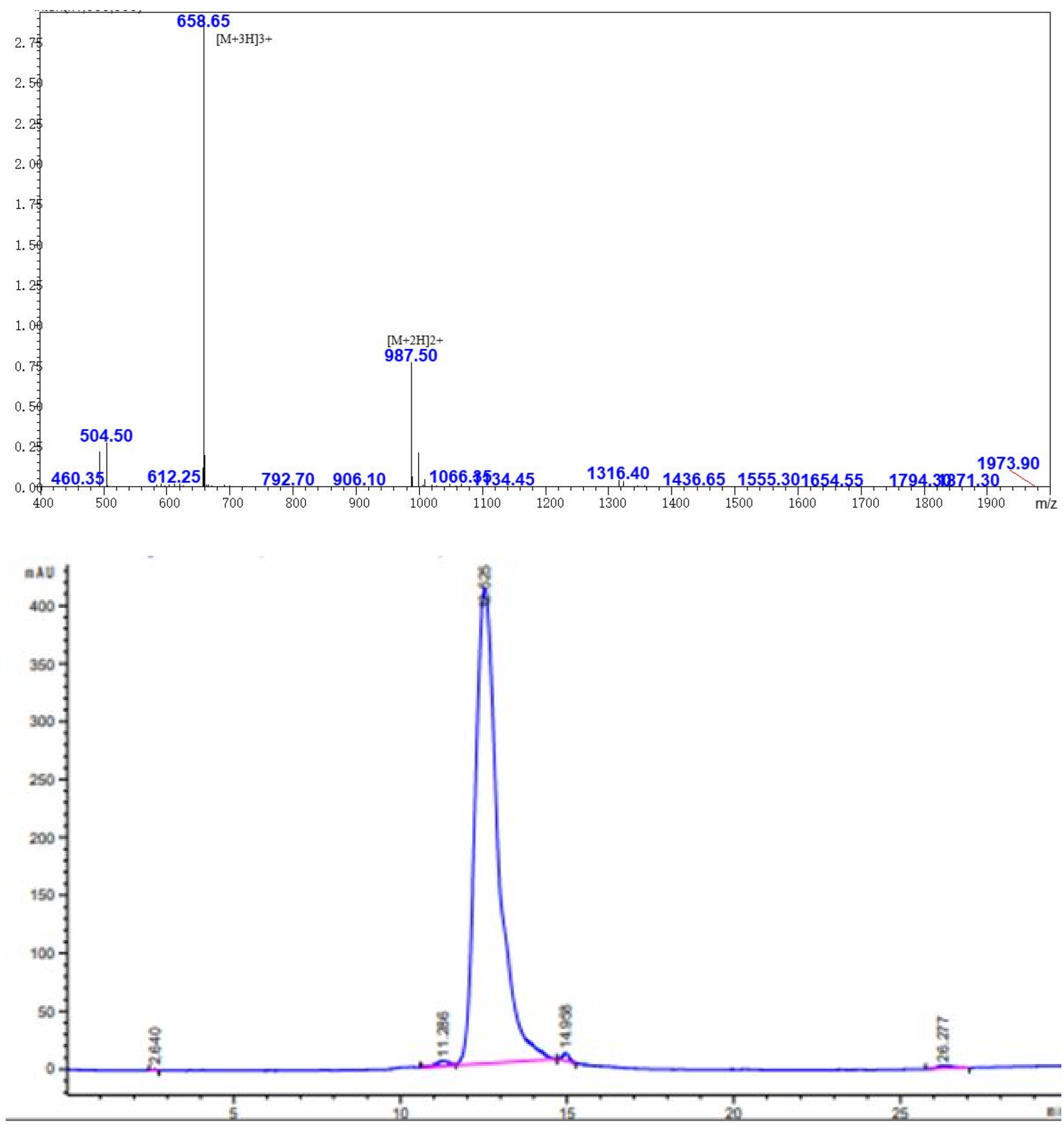

Figure S4. MS spectra (upper panel) and the RP-HPLC chromatogram (lower panel) of purified P17.

__Table S1. Consolidated list of the MIC values of all peptides in the library.__

Peptides	MIC (µM)									HL_{50} (µM)	R_t (mins)
	Bacteria								Fungi		
	Gram negative			Gram positive							
	PROTEOBACTERIA			ACTINO-BACTERIA			FIRMICUTES		ASCO-MYCOTA		
	PA	RM	SP	MY	DN	KR	MC	BL	HR		
**P1**			**0.9**	**0.9**	**0.9**	**1.9**	**0.9**	**0.9**		**≥ 200**	**13.067**
P2		0.9	0.9		0.9	0.9	1.9	0.9			12.624
**P3**		**1.9**	**1.9**	**0.9**	**0.9**		**0.9**	**1.9**		**≥ 200**	**12.731**
**P4**	**7.8**	**3.9**	**0.9**	**0.9**	**0.9**	**0.9**	**0.9**	**0.9**		**10***	**12.891**
P5	31.25		0.9	0.9	0.9	0.9		0.9	62.5		12.045
P6	7.8		15.625	0.9	0.9	0.9		0.9	0.9		12.299
P7	31.25	0.9	0.9	0.9				0.9	1.95		12.156
P8	15.625		3.91	0.9	0.9			1.9	31.25		12.168
P9	31.25		7.8	0.9	0.9		0.9	3.9			12.610
P10	31.25		7.8					3.9			12.417
**P11**	**1.9**		**1.9**	**0.9**	**0.9**		**0.9**	**1.9**		**≥ 200**	**12.875**
P12	7.8		0.9	0.9	0.9		0.9	3.9			12.903
P13	7.8		0.9	0.9		3.9	3.9	3.9	7.8		12.084
P14			0.9			0.9		3.9			12.279
P15			0.9				0.9	0.9			14.327
P16			0.9	0.9		1.9	125	0.9			14.075
**P17**	**3.8**		**0.9**	**0.9**	**0.9**	**1.9**	**1.9**	**0.9**	**3.8**	**≥ 200**	**12.625**
P18			0.9	0.9	0.9	0.9	31.25	1.9			13.314
P19			0.9	0.9	0.9	3.9		1.9			13.667

The abbreviations for microbial clusters remain the same as mentioned previously.
** P4 shows comparatively higher cytotoxicity.*

Despite showing promising MIC values, some peptides showed lesser activity when incubated in fuel environment, which are enlisted below in Table S4.

Testing the efficacy of the engineered peptides against lab-grown microbial co-cultures.

Since microbially contaminated fuel would harbour diverse microbial growth, this experimental setup is aimed to understand the effectiveness of the peptides against lab-grown microbial cocultures.

B. licheniformis (BL, Phylum – Firmicutes, Gram-positive bacteria), *D. nishinomiyaensis* (DN, Phylum – Actinobacteria, Gram-positive bacteria), *S. paucimobilis* (SP, Phylum – Proteobacteria, Gram-negative bacteria) and *H. resinae* (HR, Phylum – Ascomycota) were chosen to prepare *in vitro* microbial cocultures.

Similar growth conditions were maintained as described in the "Materials and methods" in the main manuscript. Secondary cultures were grown by inoculating fresh nutrient medium from the overnight grown primary cultures. Aliquots were isolated from the log phase of pure cultures and inoculated into fresh medium at equal proportions (v/v). Multiple combinations of microbial cocultures were prepared and allowed to grow overnight (or until visible growth is seen) at 30°C under aerobic conditions. The combinations of microbial co-cultures are described in the table S1.

Table S2. Schematic representation of the microbial cocultures grown in lab.

(The microbial abbreviations remain similar as mentioned in Table 1).

	Bacterial clusters			Fungal clusters
	Gram-positive		Gram-negative	
	Actinobacteria/DN	Firmicutes/BL	Proteobacteria/SP	Ascomycota/HR
Bacterial clusters				
Gram-positive				
Actinobacteria/DN		**DN + BL**	**DN +SP**	**DN + HR**
Firmicutes/BL				**BL+HR**
Gram-negative				
Proteobacteria/SP				**SP + HR**
Fungal clusters				
Ascomycota/HR				
		**HR + BL + SP**		
**HR + BL + DN**				
	**HR + DN + SP**			

Once confluent growth of the microbial mixed cultures was observed, cell aliquots were plated on nutrient agar plates and checked for the presence of live cells using light microscopy.

(a)

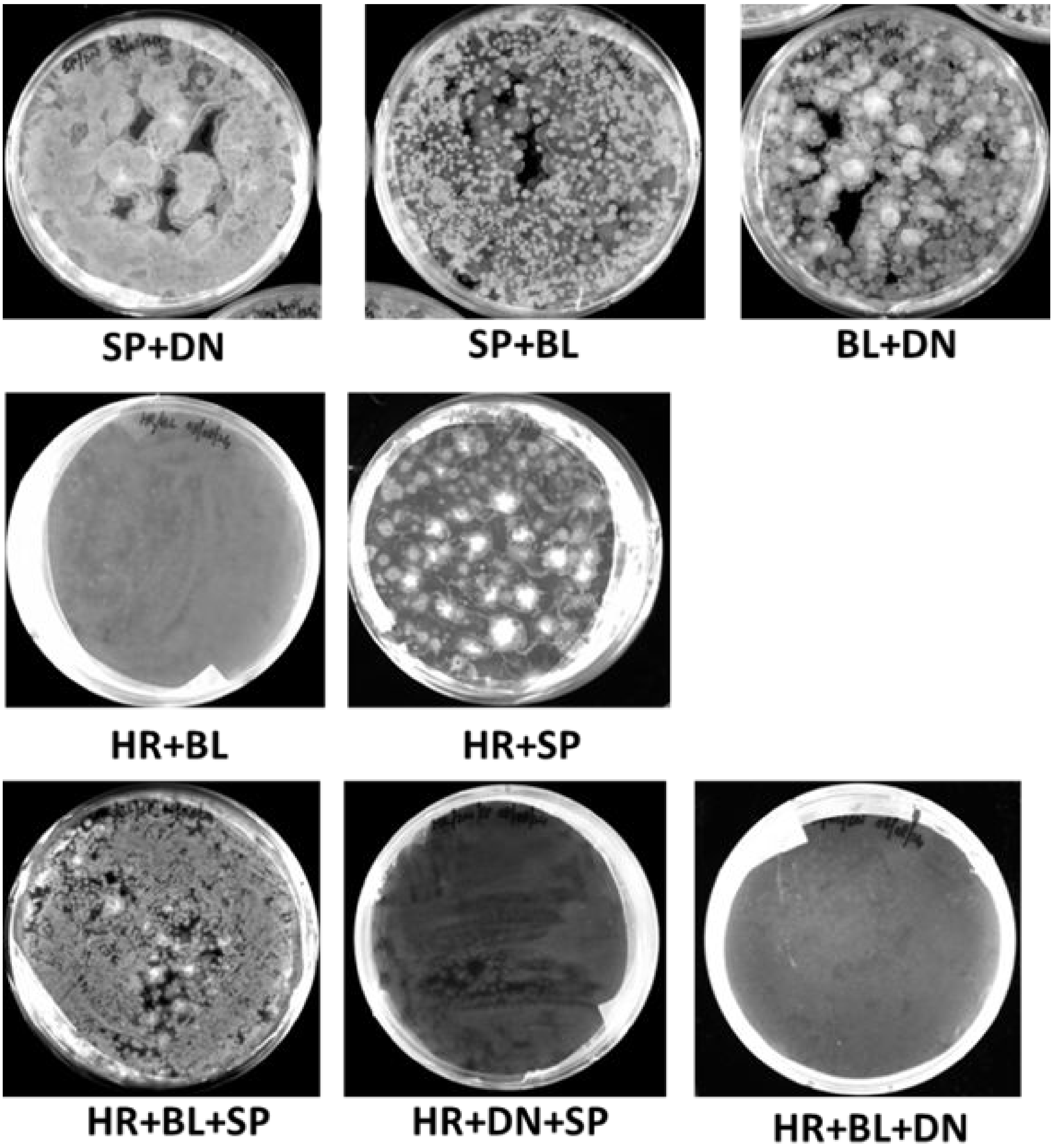

(b)

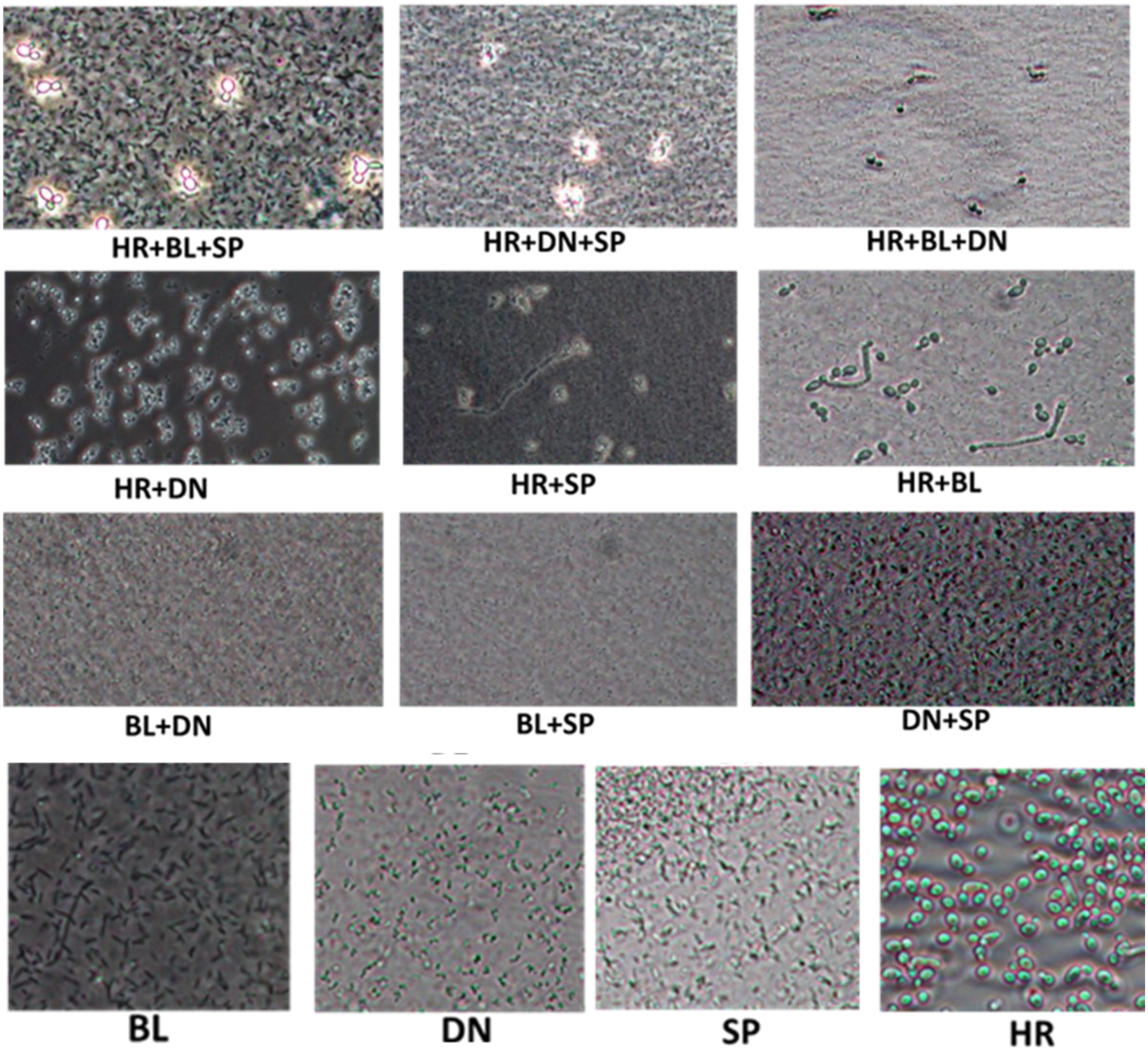

Figure S5. Tracking the growth of microbial cocultures on nutrient agar plates and using light microscopy. (a) The plates show confluent growth of the microbes in mixed culture conditions; each image is denoted with the respective microbes of the coculture in initials. (b) The three upper panels show the light microscopy images depicting live individual cells in the mixed lab grown cultures and the lowermost panel shows the light microscopy images of the individual cells.

Antimicrobial assay to check the efficacy of the peptides against the microbial cocultures.

Peptide solutions were added to the punctured holes on the solid agar plates incubated with the microbial cocultures to check the effectiveness of the best performing peptides against them. The peptides showed considerable effectiveness against the microbial cocultures, as depicted in the table S2(a). The MIC of these peptides against the microbial cocultures were determined using the method as described earlier in the main manuscript. The MIC value would give an insight into the efficacy of these peptides in controlling the growth of microbial cocultures.

Table S3. Determining the effectiveness of the engineered peptides against the mixed microbial cultures.

**(a) Effective peptides against the microbial cocultures as identified from the disc diffusion assay.** (“+” indicates the capacity of the peptides to kill the microbial cells)				
	P1	P3	P11	P17
BL+DN	+	+	+	+
SP+BL		+	+	+
DN+SP	+	+	+	+
HR+SP	+	+	+	+
HR+DN			+	+
HR+BL	+	+	+	+
HR+BL+SP	+	+	+	+
HR+BL+DN	+	+	+	+
HR+DN+SP	+	+	+	+
**(b)MIC of the best performing peptides against the microbial cocultures.**				
Microbial clusters/ peptides	P1	P3	P11	P17
	MIC (μM)			
BL+DN	15	30	60	1.87
SP+BL	3.7	1.87	120	7.5
DN+SP	3.7	3.7	30	15
HR+SP	0.9	0.9	30	7.5
HR+DN	7.5	0.9	1.87	0.9
HR+BL	3.7	0.9	3.7	7.5
HR+BL+SP	7.5	3.7	1.87	0.9
HR+BL+DN	7.5	7.5	1.87	0.9
HR+DN+SP	60	15	3.7	7.5

The novel engineered peptides, namely, P3, P11 and P17 showed significant resistance to the microbial cocultures grown in the laboratory. The *in vitro* method pursued to understand the effect of these peptides against the fuel contaminating microbial clusters, forms the foundation to development mitigation strategies against real-time contaminated conditions in fuel systems.

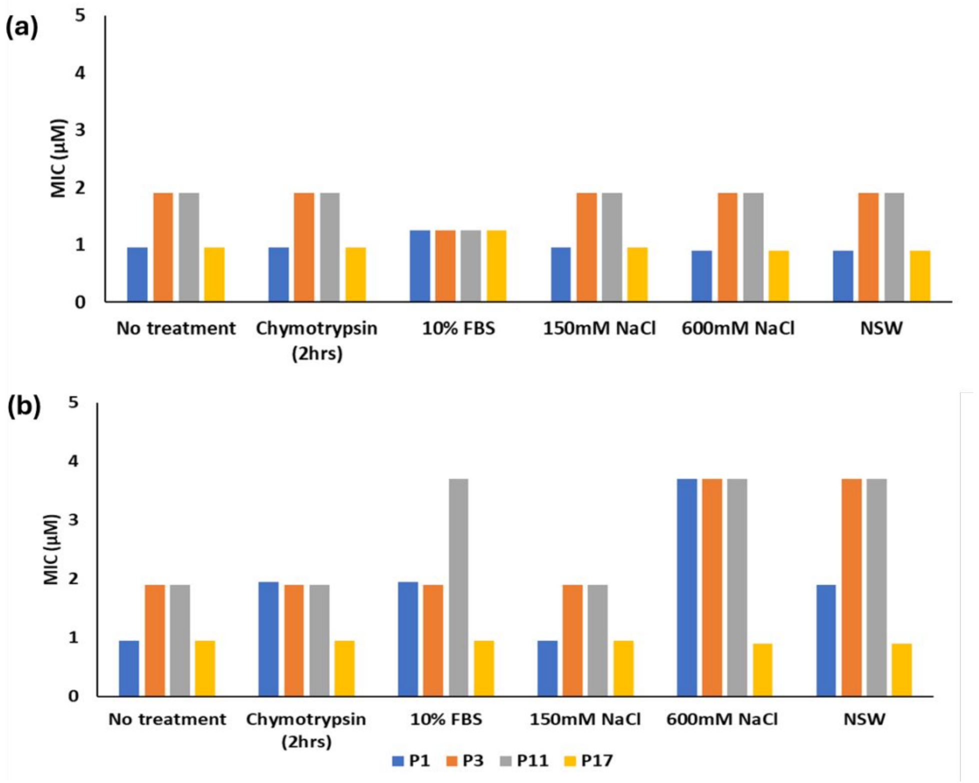

Figure S6. Stability analyses of the engineered peptides. Stability analyses of P1, P3, P11 and P17 in the presence of protease (2 h incubation), 10% FBS, 150 mM NaCl (biological condition), 600 mM NaCl and natural seawater (NSW) by determining peptide efficacy against (a) Gram-positive bacteria (*B. licheniformis*) and (b) Gram-negative bacteria (*S. paucimobilis*). The MIC of the peptides remains within the permissible range of ≤4µM under different treatments, when compared with peptides in the absence of protease, FBS and salt.

Assessing the hydrocarbon uptake capacity of the microbial clusters

The hydrocarbon uptake capacity of selected microbial clusters was assessed using Bushnell-Hass (BH) medium which contains salts such as $MgSO_4.7H_2O$, Ca $(NO_3)_2.4H_2O$, $(NH_4)_6Mo_7O_{24}.4H_2O$, KH_2PO_4, K_2HPO_4, and $(NH_4)_2SO_4$. Filter-sterilized fuel was added to the BH medium, followed by addition of microbial inoculum. The microbes were incubated at 30°C under aerobic conditions, and their growth was monitored by measuring optical density at 600 nm (OD_{600}) over 30 days, at regular intervals. Since the fuel hydrocarbon was the sole nutrient source in the experimental set up, microbial growth indicated their ability to utilize it. Emulsification of the microbes in the fuel phase was also visually observed to determine the onset and extent of microbial contamination in fuel. Plating of the samples was done to ensure viability of the cells on nutrient agar plates (Hu et al., 2020).

This section of the study aims to evaluate the ability of microbes to utilise fuel as their nutrient source and the efficacy of the engineered peptides in inhibiting the growth of microbial clusters in fuel environment. Two types of simulated fuel systems were created, i.e., (1) Bushnell media (40ml) supplemented with sterilized fuel (2ml) as described by Dong Hu et.al (2019) with slight modifications and (2) fuel system comprising 1:1 (v/v) aqueous layer (composed of BH medium(Hu et al., 2020), seawater or de-ionised water) and the solvent/fuel layer (comprising jet fuel). BH medium is a minimal growth medium which contains only essential salts, rendering it suitable for isolating and studying microorganisms that utilize hydrocarbons. The introduction of the aqueous layer in the experimental set up is to simulate water-contaminated fuel systems, where the microbial load is usually concentrated in the water layer deposited at the bottom of the tanks (Passman, 2013). For the second set up, inoculum was added to the aqueous layer and the microbial growth in both setups was monitored by measuring optical density at 600 nm at various time intervals up to 30 days (Figure S2). For both the set ups, all the tested microbes exhibited steady growth, whereas *P. aeruginosa* and *H. resinae* demonstrated rapid and confluent growth over time. These results indicate that these microbial strains can effectively utilize fuel hydrocarbons for their growth, as this was the only nutrient source.

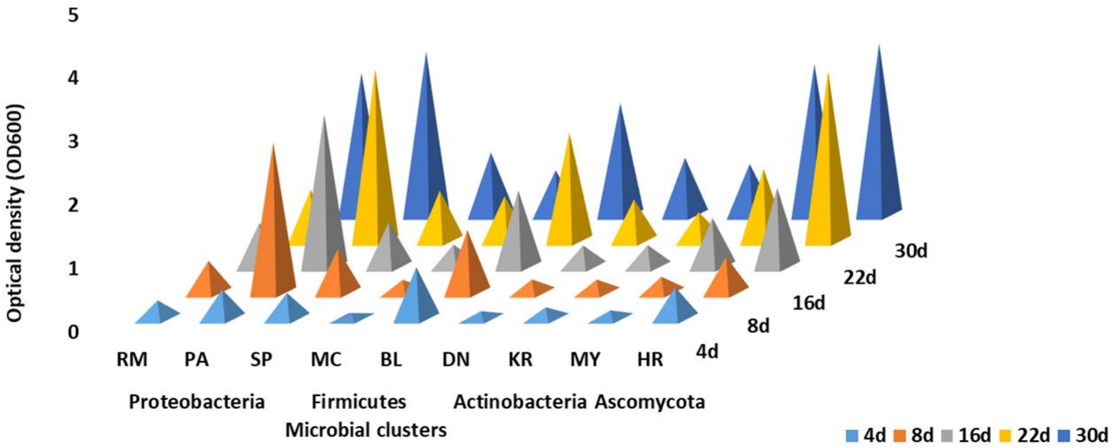

Figure S7. **Assessing the fuel hydrocarbon uptake capacity of the microbial clusters**. Optical density at 600 nm was measured to check the viability of the microbial cultures growing in the fuel system. The study showed that the spiked microorganisms could utilize the fuel hydrocarbons as the nutrient source for sustenance over the experimental duration.

Table S4. Summarizing the broad-spectrum activity of the peptides, considering the MIC value/ efficacy to control microbial growth upon fuel co-incubation

**Peptides**	**Bacterial clusters**								**Fungal clusters**	
	**Actinobacteria**			**Firmicutes**		**Proteobacteria**			**Asco-mycota**	**Score**
	**MY**	**KR**	**DN**	**MC**	**BL**	**RM**	**SP**	**PA**	**HR**	
**P1**	+	+	+	+	+	**X**	+	**X**	**X**	**6**
P2	X	+	Fuel x	+	+	Fuel x	+	X	X	2
**P3**	+	**X**	+	+	+	+	+	**X**	**X**	**6**
**P4**	+	+	+	+	+	+	+	**X**	**X**	**7**
P5	+	Fuel x	+	X	+	X	+	X	X	3
P6	+	Fuel x	Fuel x	X	Fuel x	X	X	X	Fuel x	-3
P7	+	X	X	X	+	+	+	X	Fuel x	3
P8	+	X	Fuel x	X	+	X	+	X	X	2
P9	+	X	+	+	+	X	X	X	X	4
P10	X	X	X	X	+	X	X	X	X	1
**P11**	+	**X**	+	+	+	**X**	+	+	**X**	**6**
P12	X	X	+	+	+	X	+	X	X	4
P13	+	Fuel x	X	+	+	X	+	X	Fuel x	2
P14	X	Fuel x	X	X	+	X	+	X	X	1
P15	X	X	X	+	+	X	+	X	X	3
P16	+	+	X	X	+	X	+	X	X	4
**P17**	+	+	+	+	+	**X**	+	+	+	**8**
P18	+	+	Fuel x	X	+	X	+	X	X	3
P19	+	Fuel x	+	X	+	X	+	X	X	3

Peptides identified based on the scoring matrix, where (+) =1 (implying peptide activity in fuel environment); (X) = 0 (implying no activity in the desired range, indicated by microbial proliferation); Fuel x = -1(implying no activity in fuel environment).

Clearly, P17 is the best performing peptide *in vitro* across diverse microbial phyla as well as when incubated in the fuel environment. Based on the activity of the selected peptides, the best

four peptides, P3, P4, P11 and P17, along with the template P1, were tested for their cytotoxicity. As mentioned in the main manuscript, P4 showed higher cytotoxicity and was discontinued from further applicative studies. A consolidated list of the peptides, P1, P3, P11 and P17 comprising the respective MIC values, cytotoxicity and retention time is already given in the main manuscript (Table 4).

Table S5. **Effects of engineered peptides on spiked microorganisms in the fuel environment.**

		Peptides/Days of incubation															
		P1				P3				P11				P17			
		7d	14d	21d	30d	7d	14d	21d	30d	7d	14d	21d	30d	7d	14d	21d	30d
Bacterial clusters																	
Gram-positive																	
Actino-bacteria	MY	+	+	+	+	+	+	+	+	+	+	+	+	+	+	+	+
	KR	+	+	+	+									+	+	+	+
	DN	+	+	+	+	+	+	+	+	+	+	+	+	+	+	+	+
Firmicutes	MC	+	+	+	+	+	+	+	+	+	+	+	+	+	+	+	+
	BL	+	+	+	+	+	+	+	+	+	+	+	+	+	+	+	+
Gram-negative																	
Proteo-bacteria	RM					+	+	+									
	SP	+	+	+	+	+	+	+	+	+	+	+	+	+	+	+	+
	PA									+	+	+		+	+	+	
Fungal clusters																	
Asco-mycota	HR													+	+		

"+" denotes the activity of the peptides, i.e. the microbial growth was restricted.

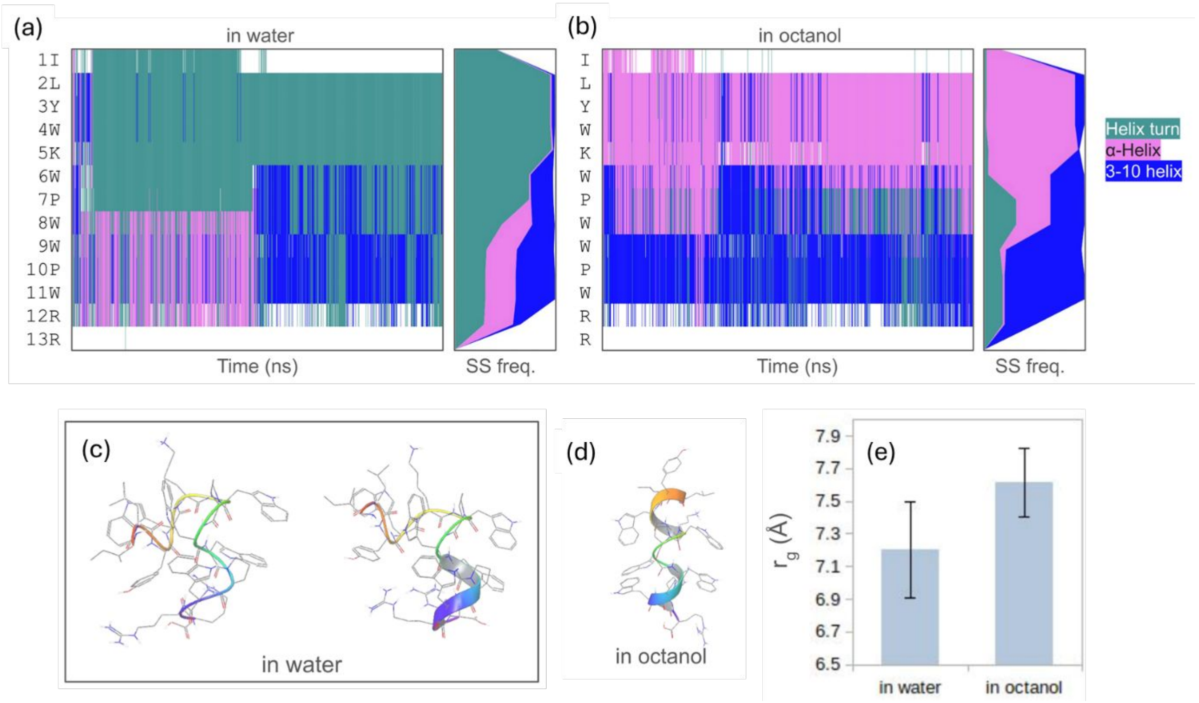

***Figure S8*. Secondary structural propensities of the peptide P17 in water and octanol environments. (a)** Residue-wise secondary structural propensities of P17 over 100 ns in water environment. Helix turn (turquoise), α-helix (pink) and 3_{10} helix (blue) conformations were observed. **(b)** Residue-wise secondary structural propensities of P17 in octanol. **(c)** Representative structure of P17 in water shows mostly turns with occasional α or 3_{10} helix near the C-terminal. The backbone is shown in ribbon representation coloured in rainbow from C- to N-terminal. **(d)** Representative structure of P17 in octanol shows elongated helical conformation. **(e)** Radius of gyration (r_g) of P17 in water and in octanol.

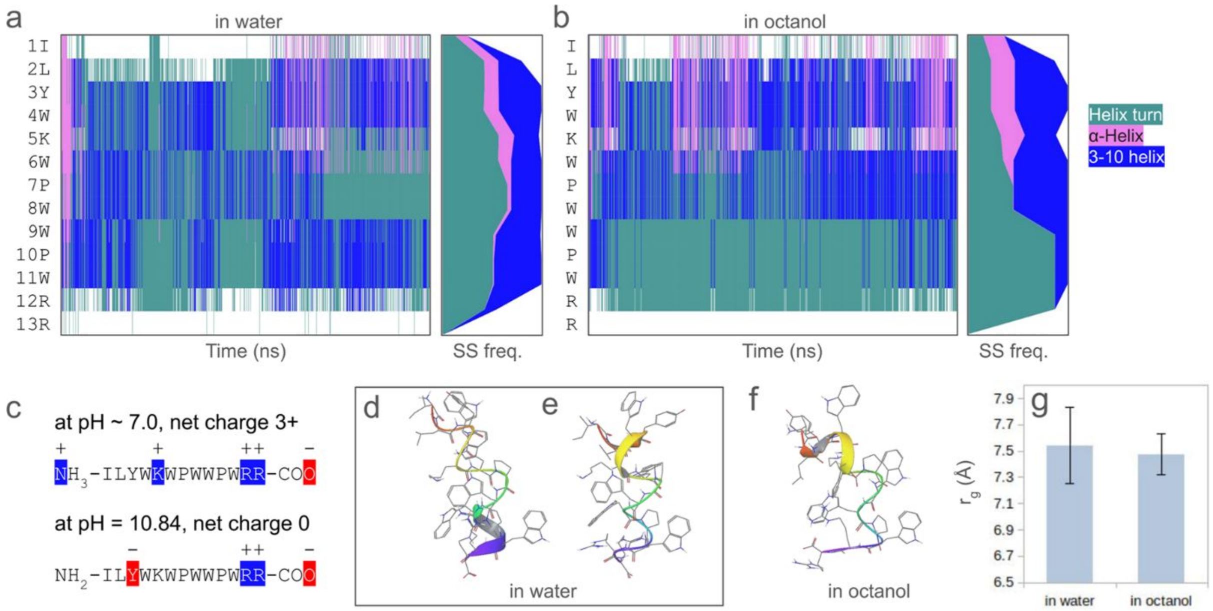

***Figure S9*. Secondary structural propensities of the charge neutral P17 (at pI) in water and octanol environments. (a)** Residue-wise secondary structural propensities of P17 over 100 ns in water environment. Helix turn (turquoise), α-helix (pink) and 3_{10} helix (blue) conformations were observed. **(b)** Residue-wise secondary structural propensities of P17 in octanol. **(c)** Comparison of the protonation states of different amino acid residues of P17 at pH 7.0 and at pI, respectively. **(d)** Representative structure of charge neutral P17 in water shows relatively extended structure with propensity towards 3_{10} helix. **(e)** The backbone is shown in ribbon representation coloured in rainbow from C- to N-terminal. **(f)** Representative structure of charge neutral P17 in octanol shows elongated helical conformation. **(g)** Radius of gyration (r_g) of charge neutral P17 in water and in octanol.

Table S6. **Thermodynamics of P17 in membrane.**

**with (type)**	**in membrane (*kcal/mol*)**
Solvent (Coulomb)	-228.33±100.59
Solvent (vdW)	9.62±7.70
**Solvent (total) [1]**	**-218.70±100.89**
Ion (Coulomb)	20.66±28.85
Ion (vdW)	-0.02±0.09
**Ion (total)**	**20.64±28.85**
Self (Coulomb)	302.16±14.59
Self (vdW)	11.30±5.95
Self (bond)	56.69±6.14
Self (angle)	150.65±8.90
Self (torsion)	71.40±6.45
**Self (total) [2]**	**592.21±20.17**
Membrane (Coulomb)	-625.56±131.45
Membrane (vdW)	-134.10±9.22
**Membrane (total) [3]**	**-759.66±131.78**
**ΔGsol [1+2+3]**	**-386.15±167.18**

***Table S7.* Thermodynamics of solvation of P17 in water and octanol.**

	**Charged**		**Neutral (at pI)**	
**with (type)**	**in water**	**in octanol**	**in water**	**in octanol**
Solvent (Coulomb)	-524.04±53.32	-293.65±28.78	-625.61±39.22	-355.04±32.27
Solvent (vdW)	-63.74±10.12	-142.96±9.18	-55.07±10.36	-138.61±11.05
**Solvent (total) [1]**	**-587.78±53.34**	**-436.61±30.21**	**-680.68±37.76**	**-493.65±31.23**
Ion (Coulomb)	-24.92±44.61	-216.99±28.35	–	–
Ion (vdW)	-0.43±1.23	1.15±3.05	–	–
**Ion (total)**	**-25.34±45.02**	**-215.84±27.17**	–	–
Self (Coulomb)	172.63±13.60	177.52±9.56	197.86±17.05	122.27±19.60
Self (vdW)	-7.09±8.63	1.03±7.17	3.33±7.37	11.58±7.20
Self (bond)	55.10±5.88	55.57±6.22	56.25±6.20	56.80±6.19
Self (angle)	141.56±9.30	143.14±8.77	144.72±8.89	146.28±8.44
Self (torsion)	69.34±6.99	68.06±6.70	70.66±7.15	70.30±6.65
**Self (total) [2]**	**431.54±20.72**	**445.31±17.42**	**472.81±21.68**	**407.22±21.68**
**ΔG_{sol} [1+2]**	**-156.24±58.09**	**8.70±34.87**	**-207.86±43.54**	**-86.42±38.01**

Table S8. **Computed pI and log D at pH 7.4 of the peptides.**

Peptide	pI	log D at pH 7.4
P1	11.04	-4.31
P3	10.86	-3.73
P11	10.56	-4.41
P17	10.84	-3.24

References

Hu, D., Zeng, J., Wu, S., Li, X., Ye, C., Lin, W., & Yu, X. (2020). A survey of microbial contamination in aviation fuel from aircraft fuel tanks. *Folia Microbiologica*, *65*(2), 371–380. https://doi.org/10.1007/s12223-019-00744-w

Passman, F. J. (2013). Microbial contamination and its control in fuels and fuel systems since 1980 - a review. *International Biodeterioration and Biodegradation*, *81*, 88–104. https://doi.org/10.1016/j.ibiod.2012.08.002
